# Molecular and Translational Classifications of DAMPs in Immunogenic Cell Death

**DOI:** 10.3389/fimmu.2015.00588

**Published:** 2015-11-20

**Authors:** Abhishek D. Garg, Lorenzo Galluzzi, Lionel Apetoh, Thais Baert, Raymond B. Birge, José Manuel Bravo-San Pedro, Karine Breckpot, David Brough, Ricardo Chaurio, Mara Cirone, An Coosemans, Pierre G. Coulie, Dirk De Ruysscher, Luciana Dini, Peter de Witte, Aleksandra M. Dudek-Peric, Alberto Faggioni, Jitka Fucikova, Udo S. Gaipl, Jakub Golab, Marie-Lise Gougeon, Michael R. Hamblin, Akseli Hemminki, Martin Herrmann, James W. Hodge, Oliver Kepp, Guido Kroemer, Dmitri V. Krysko, Walter G. Land, Frank Madeo, Angelo A. Manfredi, Stephen R. Mattarollo, Christian Maueroder, Nicolò Merendino, Gabriele Multhoff, Thomas Pabst, Jean-Ehrland Ricci, Chiara Riganti, Erminia Romano, Nicole Rufo, Mark J. Smyth, Jürgen Sonnemann, Radek Spisek, John Stagg, Erika Vacchelli, Peter Vandenabeele, Lien Vandenberk, Benoit J. Van den Eynde, Stefaan Van Gool, Francesca Velotti, Laurence Zitvogel, Patrizia Agostinis

**Affiliations:** ^1^Cell Death Research and Therapy Laboratory, Department of Cellular Molecular Medicine, KU Leuven – University of Leuven, Leuven, Belgium; ^2^Equipe 11 Labellisée Ligue Contre le Cancer, Centre de Recherche des Cordeliers, Paris, France; ^3^U1138, INSERM, Paris, France; ^4^Université Paris Descartes, Sorbonne Paris Cité, Paris, France; ^5^Université Pierre et Marie Curie, Paris, France; ^6^Gustave Roussy Comprehensive Cancer Institute, Villejuif, France; ^7^U866, INSERM, Dijon, France; ^8^Faculté de Médecine, Université de Bourgogne, Dijon, France; ^9^Centre Georges François Leclerc, Dijon, France; ^10^Department of Gynaecology and Obstetrics, UZ Leuven, Leuven, Belgium; ^11^Laboratory of Gynaecologic Oncology, Department of Oncology, Leuven Cancer Institute, KU Leuven, Leuven, Belgium; ^12^Department of Microbiology, Biochemistry, and Molecular Genetics, University Hospital Cancer Center, Rutgers Cancer Institute of New Jersey, New Jersey Medical School, Newark, NJ, USA; ^13^Laboratory of Molecular and Cellular Therapy, Vrije Universiteit Brussel, Jette, Belgium; ^14^Faculty of Life Sciences, University of Manchester, Manchester, UK; ^15^Department of Internal Medicine 3 – Rheumatology and Immunology, Friedrich-Alexander-University Erlangen-Nurnberg, Erlangen, Germany; ^16^Department of Experimental Medicine, Sapienza University of Rome, Rome, Italy; ^17^de Duve Institute, Université Catholique de Louvain, Brussels, Belgium; ^18^Department of Radiation Oncology, University Hospitals Leuven, KU Leuven – University of Leuven, Leuven, Belgium; ^19^Department of Biological and Environmental Science and Technology, University of Salento, Salento, Italy; ^20^Laboratory for Molecular Biodiscovery, Department of Pharmaceutical and Pharmacological Sciences, KU Leuven – University of Leuven, Leuven, Belgium; ^21^Sapienza University of Rome, Rome, Italy; ^22^SOTIO, Prague, Czech Republic; ^23^Department of Immunology, 2nd Faculty of Medicine, University Hospital Motol, Charles University, Prague, Czech Republic; ^24^Department of Radiation Oncology, Universitätsklinikum Erlangen, Erlangen, Germany; ^25^Department of Immunology, Medical University of Warsaw, Warsaw, Poland; ^26^Biotherapy and Vaccine Unit, Institut Pasteur, Paris, France; ^27^Wellman Center for Photomedicine, Massachusetts General Hospital, Boston, MA, USA; ^28^Cancer Gene Therapy Group, Transplantation Laboratory, Haartman Institute, University of Helsinki, Helsinki, Finland; ^29^Helsinki University Hospital Comprehensive Cancer Center, Helsinki, Finland; ^30^TILT Biotherapeutics Ltd., Helsinki, Finland; ^31^Recombinant Vaccine Group, Laboratory of Tumor Immunology and Biology, National Cancer Institute, National Institutes of Health, Bethesda, MD, USA; ^32^Metabolomics and Cell Biology Platforms, Gustave Roussy Comprehensive Cancer Institute, Villejuif, France; ^33^Pôle de Biologie, Hôpital Européen Georges Pompidou, AP-HP, Paris, France; ^34^Department of Women’s and Children’s Health, Karolinska University Hospital, Stockholm, Sweden; ^35^Molecular Signaling and Cell Death Unit, Inflammation Research Center, VIB, Ghent, Belgium; ^36^Department of Biomedical Molecular Biology, Ghent University, Ghent, Belgium; ^37^Molecular ImmunoRheumatology, INSERM UMRS1109, Laboratory of Excellence Transplantex, University of Strasbourg, Strasbourg, France; ^38^Institute of Molecular Biosciences, NAWI Graz, University of Graz, Graz, Austria; ^39^BioTechMed Graz, Graz, Austria; ^40^IRRCS Istituto Scientifico San Raffaele, Università Vita-Salute San Raffaele, Milan, Italy; ^41^Translational Research Institute, University of Queensland Diamantina Institute, University of Queensland, Wooloongabba, QLD, Australia; ^42^Laboratory of Cellular and Molecular Nutrition, Department of Ecological and Biological Sciences, Tuscia University, Viterbo, Italy; ^43^Department of Radiation Oncology, Klinikum rechts der Isar, Technische Universität München, Munich, Germany; ^44^Department of Medical Oncology, University Hospital, Bern, Switzerland; ^45^INSERM, U1065, Université de Nice-Sophia-Antipolis, Centre Méditerranéen de Médecine Moléculaire (C3M), Équipe “Contrôle Métabolique des Morts Cellulaires”, Nice, France; ^46^Department of Oncology, University of Turin, Turin, Italy; ^47^Immunology in Cancer and Infection Laboratory, QIMR Berghofer Medical Research Insitute, Herston, QLD, Australia; ^48^School of Medicine, University of Queensland, Herston, QLD, Australia; ^49^Department of Paediatric Haematology and Oncology, Children’s Clinic, Jena University Hospital, Jena, Germany; ^50^Centre de Recherche du Centre Hospitalier de l’Université de Montréal, Institut du Cancer de Montréal, Faculté de Pharmacie, Université de Montréal, Montreal, QC, Canada; ^51^Laboratory of Pediatric Immunology, Department of Microbiology and Immunology, KU Leuven – University of Leuven, Leuven, Belgium; ^52^Ludwig Institute for Cancer Research, de Duve Institute, Université Catholique de Louvain, Brussels, Belgium; ^53^Department of Ecological and Biological Sciences, Tuscia University, Viterbo, Italy; ^54^University of Paris Sud, Le Kremlin-Bicêtre, France; ^55^U1015, INSERM, Villejuif, France; ^56^Center of Clinical Investigations in Biotherapies of Cancer (CICBT) 507, Villejuif, France

**Keywords:** anti-tumor immunity, immunogenicity, immunotherapy, molecular medicine, oncoimmunology, patient prognosis, translational medicine

## Abstract

The immunogenicity of malignant cells has recently been acknowledged as a critical determinant of efficacy in cancer therapy. Thus, besides developing direct immunostimulatory regimens, including dendritic cell-based vaccines, checkpoint-blocking therapies, and adoptive T-cell transfer, researchers have started to focus on the overall immunobiology of neoplastic cells. It is now clear that cancer cells can succumb to some anticancer therapies by undergoing a peculiar form of cell death that is characterized by an increased immunogenic potential, owing to the emission of the so-called “damage-associated molecular patterns” (DAMPs). The emission of DAMPs and other immunostimulatory factors by cells succumbing to immunogenic cell death (ICD) favors the establishment of a productive interface with the immune system. This results in the elicitation of tumor-targeting immune responses associated with the elimination of residual, treatment-resistant cancer cells, as well as with the establishment of immunological memory. Although ICD has been characterized with increased precision since its discovery, several questions remain to be addressed. Here, we summarize and tabulate the main molecular, immunological, preclinical, and clinical aspects of ICD, in an attempt to capture the essence of this phenomenon, and identify future challenges for this rapidly expanding field of investigation.

## Introduction and Historical Background

Augmenting the immunogenicity of cancer cells to improve the efficacy of cancer therapy is a paradigm that has gained significant momentum over the past 5 years (1–5). Researchers have realized that besides therapeutically exploiting innate or adaptive immune cells directly (e.g., through dendritic cell (DC)-based vaccines or adoptive T-cell transfer) and/or improving the effector functions of T cells (through checkpoint-blocking therapies), cancer cells also need to be made immunogenic (1, 4, 6, 7). This has diverted attention toward studying the interface between stressed or dying cancer cells and the immune system, in the hope of efficiently exploiting it for therapeutic purposes (1).

Early indications regarding immune system-driven tumor control emerged in the eighteenth century, when feverish infections in cancer patients were circumstantially associated with tumor remission (8). The first evidence that immunotherapy can be applied to achieve tumor regression emerged from the work of William Coley, who in the 1890s achieved tumor regression in some sarcoma/lymphoma patients upon the intra-tumoral injection of streptococcal cultures (provided by Robert Koch) (8, 9). In the following 43 years, Coley injected nearly 900 (mostly sarcoma) patients with his bacterial preparation (achieving a cure rate >10%), which later became known as “Coley’s toxin” (8, 10). However, the Coley’s toxin came under intense scrutiny owing to an elevated toxicity and some difficulties in reproducing remission rates (8). Eventually, the first experimental evidence that virus-unrelated tumors can indeed be recognized by the host immune system emerged in the 1940s, and by the 1960s, coupled with the discovery of T cells, it was proposed that the human immune system may also react against tumors (11). The ability of anticancer therapies to enhance the immunogenic potential of malignant cells gained some appreciation by the 1970s (12–14). It was recognized that if specific treatments are applied (e.g., radiotherapy, the bacillus Calmette–Guerin, or some chemotherapeutics), the immunogenicity of malignant cells increases enough to induce durable anti-tumor immunity (12–14). By the 1980s, researchers started to report more specific observations regarding the therapeutic impact of cancer cell immunogenicity, e.g., the ability of curative hyperthermia to cause the (heat-shock based) generation of circumstantial anti-tumor immunity (15), the fact that the immunogenicity of cancer cells influences patient prognosis after radiotherapy (16), and the increase in tumor immunogenicity due to hydrostatic pressure (17). However, these early studies (especially those published before the 1980s) had several issues linked to a lack in consensus. For instance, due to early controversies on the existence of tumor-associated antigens (TAAs) (11), the target of tumor-specific immune responses was unclear, and the mechanism of action of some therapies came under scrutiny. Moreover, such therapies could operate by directly modulating immune effector cells rather than improving the immunogenic potential of tumors (18). In particular, the death of cancer cells exposed to therapy was never suspected to drive anti-tumor immunity, since it was considered to be a relatively “silent” process in terms of immunogenicity (19). Moreover, the classical “self/non-self” theory was unable to explain the possibility that dying cancer cells could elicit an immune response (20).

By the early 1990s, the molecular characterization of mice and human TAAs clarified the entities targeted by anti-tumor immune responses (11). Similarly, the so-called “danger theory” started to emerge, challenging the classical model of “self/non-self” immune recognition, especially in a diseased or damaged tissue (20, 21). This model proposed that the immune recognition is not restricted to “non-self” entities, but rather discriminates between “dangerous” and “safe” entities, irrespective of source (20–22). Indeed, “dangerous” entities include pathogens as well as injured, infected, diseased and necrotic tissues, or cells undergoing non-physiological cell death which emit danger signals (or alarmins) with pro-inflammatory activity (21, 22). These danger signals are now collectively referred to as “damage-associated molecular patterns” (DAMPs) (23). DAMPs are endogenous molecules that are concealed intracellularly in normal conditions, but are exposed or released upon stress, injury, cell death, thereby becoming able to bind cognate receptors on immune cells (3, 24–27). Table 1 summarizes the most prominent DAMPs characterized to date and their mode of emission, the cell death pathway they are associated with, and their known cognate receptors. It is important to consider that not all DAMPs may act as immunogenic danger signals. Several DAMPs exist that are crucial for the maintenance of tissue homeostasis, and the avoidance of auto-immune responses, as they exert immunosuppressive effects, including phosphatidylserine (PS), annexin A1 (ANXA1), death domain 1α (DD1α), B-cell CLL/lymphoma 2 (BCL2) and some extracellular matrix-derived molecules (Table 1). Accordingly, the blockade of these anti-inflammatory DAMPs accentuates the immunogenic potential of dying cells, or renders immunogenic otherwise tolerogenic forms of cell death (28, 29). Moreover, some danger signals are not always involved in the immunogenicity of cell death, but act as “bystanders.” This is the case for heat shock protein 90 kDa alpha (cytosolic), class A member 1 (HSP90AA1, best known as HSP90) exposed on the cell surface after melphalan treatment (30). Last (but not least), several DAMPs may be subjected to post-translational modifications (e.g., oxidation, reduction, citrullination) that may potentially neutralize, increase, or change their immunogenic properties (31, 32) – a process that is still incompletely understood.

**Table 1:** **A list of prominent damage-associated molecular patterns (DAMPs) associated with cell death pathways or extracellular matrix**.

DAMPs	Localization and mode-of-emission	Relevant cell death pathway	Receptors	Reference
Annexin A1	Surface exposed or actively/passively released?	Apoptosis	FPR-1 receptor	(33)
Adenosine triphosphate	Actively or passively released	ICD, apoptosis/secondary necrosis and necrosis	P_2_Y_2_ and P_2_×_7_	(34–37)
B-cell CLL/lymphoma 2	Passive release	Necrosis	TLR2	(38)
Biglycan	Extracellular matrix	–	TLR2, TLR4, P_2_×_4_, and P_2_×_7_	(39, 40)
Calreticulin	Mostly surface exposed; sometimes passively released	ICD	CD91	(35, 41–44)
Cardiolipin	Surface exposed?	Apoptosis	?	(45, 46)
Ceramide and sphingosine-1-phosphate	Surface exposed	Apoptosis	?	(47)
Covalent/cross-linked dimer of ribosomal protein S19	Passively released?	Apoptosis	CD88	(48–51)
Carbamoyl-phosphate synthase 1	?	?	?	(52)
Cyclophilin A	Passive release	Necrosis	CD147	(53)
Cytochrome *c*	Passively released?	Secondary necrosis and necrosis?	LPG?	(54, 55)
Death domain 1α	Surface exposed	Apoptosis	DD1α	(56)
Endothelial monocyte-activating polypeptide II	Passively released?	Apoptosis	CXCR3?	(50, 57, 58)
F-actin	Passive release	Necrosis	DNGR-1/Clec9a	(59)
Fibrinogen	Extracellular matrix	–	TLR4	(40)
Fibronectin extra domain A	Extracellular matrix	–	TLR4?	(40)
Fragments of human tyrosyl tRNA synthetase	Passively released?	Apoptosis	?	(50)
Genomic DNA, mRNA, snRNPs	Passive release	Necrosis	TLR3	(3, 60, 61)
GRP78/BiP	Passive release	Necrosis, apoptosis?	?	(31)
H_2_0_2_	?	Apoptosis	?	(62)
Heat shock proteins (HSP70, HSP90, HSP60, HSP72, and GP96)	Surface exposure, active secretion, or passive release	ICD, apoptosis/secondary necrosis, necrosis	CD91, TLR2, TLR4, SREC-1 and FEEL-1	(63–67)
Heparan sulfate fragments	Extracellular matrix	–	TLR4	(40)
Hepatoma-derived growth factor	Passively released	Necrosis	?	(68)
Histones	Passively released	Necrosis	TLR-9	(69)
High-mobility group box 1	Mostly passively released; sometimes actively released	ICD, secondary necrosis and necrosis	TLR2, TLR4, RAGE and TIM3	(70–73)
High-mobility group nucleosome binding domain 1	Passive release	Necrosis	TLR4	(74)
Hyaluronan	Extracellular matrix	–	TLR2 and TLR4	(40)
IL-1α	Passive release	Necrosis	IL-1R	(75)
IL-33	Passive release	Necrosis	ST2	(3, 61)
IL-6	Passive release	Necrosis	IL-6R and GP130	(76)
Lysophosphatidylcholine	Passively released?	Apoptosis	G2A	(50, 77)
Mit DNA	Passively released	Necrosis	TLR-9	(78–80)
Monosodium urate or uric acid	Passively released	Necrosis	Purinergic receptors	(50, 81)
*N*-formylated peptides	Passively released	Necrosis	FPR-1	(78, 82–84)
Oxidation-associated molecular patterns (reactive protein carbonyls, per-oxidized phospholipids, oxidized low-density lipoprotein)	Passively released	Necrosis, Secondary necrosis	CD36, SR-A, TLR-2/4, CD14	(85–87)
Peroxiredoxin 1	Actively secreted or passively released	Apoptosis, necrosis	TLR4	(88)
Phosphatidylserine	Actively externalized on the surface	Apoptosis	TIM-1/-3/-4, BAI1, Stabilin-2, MFG-E8, C1q	(56, 89–93)
S100/calgranulin protein family members (S100A8, S100A9, S100A12/EN-RAGE)	Passively released	Necrosis	RAGE	(50, 94)
Tenascin-C	Extracellular matrix	–	TLR4?	(95)
Thrombospondin 1 and its heparin-binding domain	Passively released or surface associated	Apoptosis	α_v_β_3_ integrin	(50, 96)
Versican	Extracellular matrix	–	TLR2, TLR6, and CD14	(40)

*CD, cluster of differentiation; CLEC9A, C-type lectin domain family 9, member A; CPS-1, carbamoyl-phosphate synthase 1, mitochondrial; CXCR3, C-X-C motif receptor 3; FEEL-1/CLEVER-1, fasciclin EGF-like/common lymphatic endothelial and vascular endothelial receptor-1; FPR-1, formyl peptides receptor-1; G2A, G2 accumulation; HMGB1, high-mobility group box 1; HSP, heat shock proteins; ICD, immunogenic cell death; IL, interleukin; LPG, leucine-rich alpha-2-glycoprotein-1; MFG-E8, milk fat globule-egf factor 8 protein; Mit DNA, mitochondrial DNA; P2XR, P2X receptor; P2YR, P2Y receptor; RAGE, receptor for advanced glycation endproducts; SREC-1, scavenger receptor class f member 1; TFAM, mitochondrial transcription factor A; TIM, transmembrane immunoglobulin and mucin domain; TLR, toll-like receptor(s)*.

*Glossary (5, 19, 97): (1) *Necrosis*: primary necrosis is a form of cell death that can occur in a regulated or accidental manner, characterized by cellular swelling and rapid breakdown of the plasma membrane; (2) *Necroptosis*: necroptosis is a form of regulated cell death (RCD) manifesting with necrotic morphology and controlled by a signaling cascade involving (among other proteins) RIPK1, RIPK3, and MLKL; (3) *Apoptosis*: apoptosis is a form of RCD largely dependent on caspases activity and morphologically characterized by cell shrinkage, membrane blebbing, formation of apoptotic bodies, chromatin condensation, and systematic DNA fragmentation; (4) *Secondary Necrosis*: Secondary necrosis is a terminal process experienced by late-apoptotic cells if they are not cleared by phagocytes in time, and is characterized by general spill-over of apoptotic cellular contents*.

*“?” Unclear or not determined yet*.

**Figure 1:**
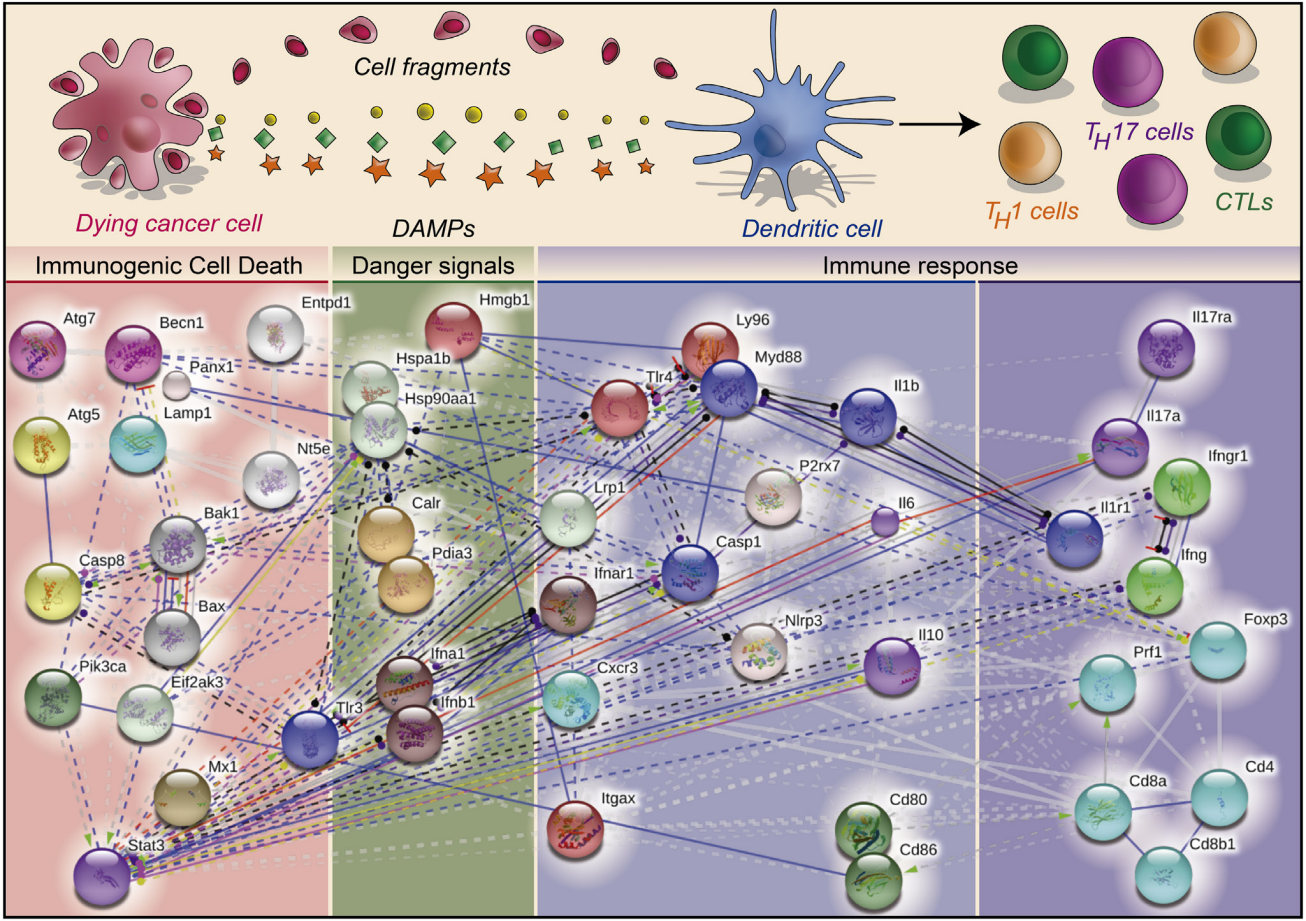
**The molecular complexity of immunogenic cell death in cancer**. Cancer cells undergoing immunogenic cell death (ICD) emit danger signals for establishing a productive interface with components of the host immune system, including dendritic cells (DCs). DCs exposed to cancer cells succumbing to ICD “prime” the adaptive arm of the immune system, consisting of various effector T-cell populations, which in turn targets therapy-resistant cancer cells. Various molecules are critical for the execution of these processes. The molecular network of ICD-relevant proteins was build using the STRING modeling database (http://string-db.org/) (126).

Despite these advances, the overall role of regulated cell death (RCD) (97) in augmenting cancer immunogenicity remained obscure. Initial observations involving the immunogenicity of cell death in the efficacy of cancer therapy were published between 1998 and 2004, when it was proposed that the non-apoptotic demise of malignant cells (within the context of the so-called “immunogenic death”) could be associated with the emission of the danger signal heat shock 70 kDa protein 1A (HSPA1A, best known as HSP70) (Table 1), enhancing the immunogenic potential of dying cancer cells *in vivo* (98, 99). The dogmatic view that only necrotic or non-apoptotic (as postulated by the “immunogenic death” concept) cancer cells are characterized by an elevated immunogenic potential started to be questioned by a series of studies published between 2005 and 2007 (41, 70, 100, 101). These publications outlined that cancer cells undergoing apoptosis in response to specific anticancer therapies are immunogenic [a subroutine termed immunogenic cell death (ICD)], as long as they emit precise DAMPs in a spatiotemporally defined fashion (26, 102, 103). Cells succumbing to ICD are sufficient for the elicitation of durable anti-tumor immune responses (1, 26, 53, 102, 104). ICD is indeed paralleled by the redirection and emission of DAMPs, owing to the stimulation of distinct danger signaling pathways occurring in synchrony with cell death signaling (103). Table 2 summarizes the main signaling pathways that play a role in the trafficking and emission of DAMPs. ICD-associated DAMPs and other immunostimulatory factors released by cells destined to undergo ICD favor the establishment of a productive interface between dying cancer cells and innate immune cells (like DCs or macrophages), thereby leading to the initiation of a therapeutically relevant adaptive immune response (Figure 1) (102, 105). In some contexts, DAMPs may regulate the function of specific innate immune cell subsets, e.g., following anthracycline treatment, extracellular adenosine triphosphate (ATP) assists in recruitment and differentiation of CD11c^+^Cd11b^+^Ly6C^high^ cells into CD11c^+^CD86^+^MHCII^+^ DCs (106); similarly, necrosis associated F-actin exposure activates an immune response by directing the dead cell debris to specifically CD8α^+^ DCs (59, 107). Indeed, DCs and other antigen-presenting cells exposed to cancer cells succumbing to ICD can then prime CD4^+^ T cells (and polarize them into T_H_1, T_H_17, or T_H_1/T_H_17-like phenotype), CD8^+^ cytotoxic T lymphocytes (CTLs) and γδ T lymphocytes against one or several TAAs (Figure 1) (102). Of note, residual cancer cells that survive ICD inducers can also show some enduring immunogenic characteristics that make them susceptible to immunological control by CTLs (108–110).

**Table 2:** **Danger signaling pathways characterized as traffickers of DAMPs**.

DAMPs	Role of ROS	Role of ER stress	Role of autophagy	Role of chaperone-mediated autophagy	Role of secretory pathway	Caspase activity	Role of lysosomes	Comments	Reference
Secreted ATP	+	+/0	+/0	0	+/0	+	+/0	Underlying pathway is highly inducer dependent	(34, 35, 111–113)
Released HMGB1	0	0	+	?	0	–	?	Mostly released passively on account of necrosis; only DT-EGF reported to cause active secretion so far	(73, 114, 115)
Secreted or surface HSP70	?	?	?	?	?	+	+	ABC transporters help in endolysosomal-secretion; HSP70 has also been reported to be secreted in an exosome surface-bound format	(116–122)
Surface CRT	+	+	−/0	+	+	+/0	?	LRP1/lipid rafts mediate surface tethering; components that positively regulate surface-CRT in an inducer-dependent fashion: ERp57, PI3K p110α, BAX/BAK, cytosolic ER-Ca^2+^, BAP31; of note, anthracycline-induced pathway of surface CRT induction has been found to be conserved from yeast to mammals	(34, 35, 111, 112, 116, 123, 124)
Surface HSP90	+	+	–	?	+	+	?	–	(30, 125)

*“+” denotes ability to positively regulate trafficking; “−” denotes ability to negatively regulate trafficking; “0” denotes confirmation of no role in regulation of trafficking and “?” denotes that the role in regulating the trafficking is unknown; “+/0” denotes positive or no role in regulation of trafficking in an inducer-dependent fashion; “−/0” denotes negative or no role in regulation of trafficking in an inducer-dependent fashion*.

*ATP, adenosine triphosphate; CRT, calreticulin; DT-EGF, epidermal growth factor receptor-targeted diphtheria toxin; ER, endoplasmic reticulum; HMGB1, high-mobility group box 1 protein; HSP, heat shock protein; LRP1, low-density lipoprotein receptor-related protein 1; ROS, reactive oxygen species*.

## Immunogenic Cell Death Inducers

Over the past few years, a number of single-agent ICD inducers have been discovered, encompassing conventional chemotherapeutics, targeted anticancer agents and various other ­biological and physicochemical therapies (18, 102, 104, 127). Table 3 summarizes single-agent ICD inducers characterized so far, as per consensus guidelines (104), and the spectra of DAMPs and other immunostimulatory signals associated with them. For combinatorial therapeutic strategies capable of achieving ICD, readers may want to refer to other recent publications (18, 128, 129). It is clear that a general structure–function relationship capable of clustering all existing ICD inducers and predicting new ones does not exist (130), an issue that makes discovering new ICD-inducing therapies based on cheminformatic analyses challenging, if not impossible. A peculiar characteristic of most, if not all, ICD inducers is their ability to induce reactive oxygen species (ROS)-based/associated endoplasmic reticulum (ER) stress, as first delineated for anthracyclines (30, 34, 35, 42, 123, 131–133). This peculiarity was exploited for the targeted discovery of hypericin-based photodynamic therapy (Hyp-PDT) – a therapeutic modality that can trigger ICD through the induction of ROS that target the ER (35, 116, 134). Along with an ever more precise characterization of the links between ROS, ER stress, and ICD induction (135, 136), it became clear that the more “focused” ER stress is, the higher the probability of inducing ICD (3, 26, 53, 137). These observations paved way for a classification system based on how ICD inducers engage ER stress for cell death and danger signaling (3, 26, 53, 138). Based on this classification, Type I ICD inducers are defined as anticancer agents that act on non-ER proteins for the induction of cell death, but promote collateral ER stress for danger signaling, thereby operating on multiple targets (3, 26, 53), while Type II ICD inducers are anticancer agents that target the ER for both cell death induction and danger signaling (3, 26, 53). Table 4 summarizes the classification of current ICD inducers into Type I and Type II, and their cell death/danger signaling targets. Such a classification suggest that while Type I ICD inducers can be discovered through various approaches (e.g., DAMP-based drug screening platforms) (130, 139), putative Type II ICD inducers can be characterized rapidly on the basis of their ability to selectively or predominantly target the ER. Recent findings comforted the purpose and usefulness of this classification system, as two novel Type II ICD inducers [i.e., Pt^II^ N-heterocyclic carbene complex (140) and Newcastle disease virotherapy (NDV) (43)] were identified based on the notion that they induce predominant ROS-based ER stress (138). Nevertheless, as more ICD inducers and features are discovered, this classification system is expected to evolve or be substituted by a more refined one.

**Table 3:** **A list of prominent single-agent immunogenic cell death (ICD) inducers in cancer and their specific associations with danger signaling and other immunostimulatory signaling**.

ICD inducers	Associated ICD-relevant DAMPs		Other immunostimulatory activities or danger signals and other comments on immunomodulatory activity	Reference
	DAMP	Stage of cell death		
Anthracyclines (epirubicin, doxorubicin, idarubicin, mitoxantrone), oxaliplatin, UVC radiation and radiotherapy	Surface CRT Surface HSP70 Secreted ATP Released HMGB1	Pre-apoptotic Mid-apoptotic Early/mid-apoptotic Post-apoptotic	Activation of Type I IFN response comprising MX-1 centered signature, consisting of IFN-α/β and CXCL10; surface exposure of mannose-6-phopshate receptor, which enables better interface with CTLs and facilitates GZMB-mediated cell death; radiotherapy is known to increase expression levels of various antigens in number of cancer models as well as induce “abscopal effect” in both preclinical and clinical models; overall *CALR* levels were predictive of prolonged OS in radiotherapy-treated lung cancer patients	(26, 42, 102, 127, 141–144)
Anti-EGFR antibody – 7A7	Surface CRT	Pre-apoptotic	–	(145)
	Surface HSP70	Early/mid-apoptotic		
	Surface HSP90	Early/mid-apoptotic		
Bleomycin	Surface CRT	Mid/post-apoptotic	Induces ambivalent immune response, i.e., all valid ICD markers but also increased Treg differentiation and, thus, a good candidate for anti-Treg combinatorial therapy	(146)
	Secreted ATP	Mid/post-apoptotic		
	Released HMGB1	Post-apoptotic		
Bortezomib	Surface HSP90	Early/mid-apoptotic	–	(26, 66, 100, 127)
	Surface CRT	Early/mid-apoptotic		
	Surface HSP70	Early/mid-apoptotic		
Oncolytic Adenovirus	Surface CRT	?	Immunogenicity of these viruses can be further increased by producing transgenic versions producing CD40L or GM-CSF	(147, 148)
	Released ATP			
	Released HMGB1			
*Clostridium difficile* toxin B	Surface CRT	Early/mid-apoptotic	–	(149)
	Released ATP	Post-apoptotic		
	Released HMGB1	Post-apoptotic		
	Released HSP70/90	Post-apoptotic		
Coxsackievirus B3 (CVB3)^#^	Surface CRT	Early-apoptotic	–	(150, 151)
	Secreted ATP	Early/mid-apoptotic		
	Released HMGB1	Post-apoptotic		
Cyclophosphamide	Surface CRT	Pre-apoptotic	Facilitates an interface between gut microbiota (leaked due to gut perforation) and host immune system thereby allowing Th17 cells-dependent anti-tumor immune responses; cyclophosphamide’s effects on anti-tumor immunity are strongly dose dependent. High doses of this chemotherapeutic can be immunosuppressive yet low or metronomic doses facilitate anti-tumor immunity through targeted depletion of Tregs/MDSCs. In ICD set-up, a low dose (100 mg/kg in mice) of cyclophosphamide was shown to exert anti-tumor immunity	(18, 152, 153)
	Released HMGB1	Post-apoptotic		
High hydrostatic pressure	Surface CRT	Early/mid-apoptotic	–	(154–156)
	Surface HSP70	Early/mid-apoptotic		
	Surface HSP90	Early/mid-apoptotic		
	Secreted ATP	Mid/post-apoptotic		
	Released HMGB1	Mid/post-apoptotic		
Hypericin-based PDT	Surface CRT	Pre-apoptotic	High accumulation of OAMPs like protein carbonyls; down-regulates CD47; induces up-regulation of various molecules associated with Type I IFN response (*IRF7*, *IRF1*, *OASL, IL18, CXCL2, IL15, IL8*) but not IFN-α secretion	(26, 30, 34, 35, 112, 116, 157)
	Surface HSP70	Pre-apoptotic		
	Surface HSP90	Pre-apoptotic		
	Secreted ATP	Pre-apoptotic		
	Released HMGB1	Post-apoptotic		
	Released HSP70/90	Post-apoptotic		
	Released CRT	Post-apoptotic		
Microwave thermal ablation	Surface CRT	?	–	(158)
	Secreted ATP			
	Released HMGB1			
Newcastle disease virus (NDV)	Surface CRT	Early/mid-necroptotic	Increases expression levels of PMEL17 antigen in glioma cells; NDV treatment has also been shown to induce “abscopal effect” in a murine melanoma model	(43, 159)
	Released HMGB1	Post-necroptotic		
Paclitaxel	Surface CRT Released HMGB1	Early/mid-apoptotic Post-apoptotic	Overall *CALR* levels were predictive of prolonged OS or PFS in paclitaxel-treated ovarian cancer patients thereby establishing clinical validity of ICD in paclitaxel treatment set-up; paclitaxel has also been reported to enhance overall antigen levels	(42, 144, 160)
Patupilone	Surface CRT	Early/mid-apoptotic	–	(128)
Photofrin-based PDT	Surface CRT	Early/mid-apoptotic	The only anticancer modality for which a comparison between DAMPs induced by *in vitro* versus *in vivo* treatment was carried out – however, none of ICD-related DAMPs were tested	(47, 161–164)
	Surface HSP70/60	Early/mid-apoptotic		
	Released HMGB1	Post-apoptotic		
	Surface ceramide	Early/mid-apoptotic		
	Surface S1P	Early/mid-apoptotic		
Pt^II^ N-heterocyclic carbene complex	Surface CRT	Pre-apoptotic	–	(140)
	Released ATP	Post-apoptotic		
	Released HMGB1	Post-apoptotic		
RIG-I-like helicases (RLH) ligand	Surface CRT	Early-apoptotic	Induces Type I IFN response	(165)
	Released HMGB1	Post-apoptotic		
	Released HSP70	Post-apoptotic		
Septacidin	Surface CRT	Pre-apoptotic	–	(139)
	Secreted ATP	Early/mid-apoptotic		
	Released HMGB1	Post-apoptotic		
Shikonin	Surface CRT	Early/mid-apoptotic	Also, causes surface exposure of GRP78 a prominent inducer of pro-tumorigenic effects; enhances overall cancer antigen levels	(160)
	Surface HSP70	Early/mid-apoptotic		
Vorinostat	Surface CRT	Early/mid-apoptotic	–	(166)
	Secreted ATP	Post-apoptotic		
	Released HMGB1	Post-apoptotic		
Wogonin	Surface CRT	Early-apoptotic	Surface-Annexin A1 is also induced by wogonin. In an ICD set-up, the role of Annexin A1 is not clear since it is a noted anti-inflammatory factor	(167)
	Released ATP	Post-apoptotic		
	Released HMGB1	Post-apoptotic		

*CRT or *CALR*, calreticulin; CTLs, cytotoxic T lymphocytes; DAMPs, damage-associated molecular patterns; EGFR, epidermal growth factor receptor; GZMB, granzyme B; HMGB1, high-mobility group box-1 protein; HSP, heat shock protein; ICD, immunogenic cell death; IFN, interferon; MDSC, myeloid-derived suppressor cells; OAMPs, oxidation-associated molecular patterns; OS, overall survival; PFS, progression-free survival*.

*Important note: It is worth noting that recently various promising candidate therapies have emerged that induce *in vitro* DAMPs relevant for ICD, e.g., Rose Bengal-based PDT (168), Docosahexaenoic acid (169), and Capsaicin (170, 171). Such agents may emerge as potent inducers of ICD in future, however, in order to establish them as inducers of ICD-like immunogenicity, it is imperative to confirm their (i.e., cancer cells treated with these agents) ability to stimulate T cells (*in vitro* or *in vivo*) and/or induce anti-cancer vaccination effect, *in vivo*, as per the consensus guidelines (104)*.

*Glossary: In the current setting, it is crucial to differentiate between the meanings of the words, “immunogenic” and “immunogenicity” as they are not supposed to have inter-changeable meanings. *Immunogenic*, derives from the word *immunogen*, which refers to any substance that can elicit an immune response; this includes, whole cells or organisms (eukaryotic or prokaryotic), specific cellular entities or specific proteins (e.g., antigens) (172). On the other hand, *immunogenicity* is a much more specific terms that is closer to *antigenicity* in operational sense, since it refers to the ability of a specific entity (e.g., an antigen or an epitope) to be recognized by the immune system through binding interactions with T or B cells, which may or may not result in an overt immunological response (4, 11)*.

*“?” Unclear or not determined yet*.

*“#” Unconfirmed anti-tumour immune responses in adaptive immune system-competent*.

**Table 4:** **Classification of ICD inducers into Type I and Type II based on their ER or non-ER-targeting *modus operandi***.

ICD inducer	Site of Cell-death inducing effects	Site of danger signaling induction	Reference
**Type I inducers – agents that induce icd through a “collateral” er stress effect**			
Anthracyclines (epirubicin, doxorubicin, idarubicin, mitoxantrone), oxaliplatin, UVC radiation and radiotherapy	Nucleus (DNA or the DNA replication machinery proteins)	ER, autophagy, pannexin channels, lysosomes	(36, 41, 70, 111, 130, 173, 174)
Anti-EGFR antibody – 7A7	Cell surface (epidermal growth factor receptor or EGFR)	ER	(145)
Bleomycin	Nucleus (causes DNA strand-breaks)	ER?	(146)
Bortezomib	Cytosol (26S proteasome or ERAD machinery; CIP2A/cancerous inhibitor of protein phosphatase 2A)	ER	(100, 175, 176)
*Clostridium difficile* toxin B	Cytoskeleton (causes cytoskeletal disruption by targeting RhoA, CDC42 and Rac1)	ER	(149, 177)
Cyclophosphamide	Nucleus (DNA)	ER	(152)
High hydrostatic pressure	Broad disrupting/denaturing effects on membranes, and proteins	ER (mitochondria?)	(154, 178)
Microwave thermal ablation	Hyperthermic ablation of cellular components	ER?	(158)
Paclitaxel, patupilone	Cytoskeleton (target microtubules thereby disrupting cytoskeletal functions)	ER	(42, 104, 179)
Photofrin-based PDT	Cellular membranes (ROS-based damage of membranes)	ER?	(180, 181)
RIG-I-like helicases (RLH) ligand	Cytosol (targets RIG-I-like helicases)	ER?	(165)
Septacidin	?	ER	(139)
Shikonin	Cytosol (tumor-specific pyruvate kinase-M2 protein)	ER	(160, 182)
Vorinostat	Nucleus/Cytosol (targets histone deacetylase)	ER?	(166)
Wogonin	Mitochondria (generates mitochondria-derived ROS)	ER	(167, 183)
**Type II inducers – agents that induce icd through a “focused” er stress effect**			
Hypericin-based PDT	ER (ROS-based damage at the ER membrane)	ER	(35, 63, 116, 181, 184, 185)
Oncolytic adenovirus	ER (ER membranes and lumen)	ER	(104, 147)
Oncolytic coxsackievirus B3 (CVB3)	ER (ER membranes and lumen)	ER	(150, 186)
Oncolytic Newcastle disease virus (NDV)	ER (ER membranes and lumen)	ER	(43, 159, 187)
Pt^II^ N-heterocyclic carbene complex	Predominantly targets ER (generates ER-directed ROS)	ER	(140)

*EGFR, epidermal growth factor receptor; ER, endoplasmic reticulum; ICD, immunogenic cell death; PDT, photodynamic therapy; ROS, reactive oxygen species*.

*“?” Unclear or not determined yet*.

Since its discovery, a plethora of molecular and immunological components responsible for ICD have been discovered (Figure 1) (26, 102, 188). Table 5 summarizes the molecular and immunological determinants of ICD characterized so far, as well as the models of ICD in which they operate (in a positive, negative or dispensable manner). Anthracyclines and oxaliplatin are the most common ICD inducers employed in experimental settings, followed by Hyp-PDT. According to current understanding, cancer cell-associated determinants of ICD can be subdivided into those that are common to all ICD inducers (i.e., “core” signaling components), and those that operate in an ICD inducer-dependent manner (i.e., “private” signaling components) (26, 189). Thus, eukaryotic translation initiation factor 2-alpha kinase 3 (EIF2AK3, best known as PERK) and the ER-to-Golgi secretory machinery are considered “core” signaling components on the cancer cell side (26, 102). Similarly, from the immune system side, a general role for (IFNγ-producing) CD4^+^ and CD8^+^ T cells has been confirmed for most, if not all, ICD inducers (Table 5). Interestingly, some components that are required for ICD induction by some agents (like autophagy for anthracyclines and oxaliplatin) (190) might be either dispensable for ICD induction by other agents, e.g., autophagy for NDV (43) and phosphorylation of eukaryotic translation initiation factor 2α (eIF2α), caspase-8 (CASP8) activation or cytosolic Ca^2+^ levels for Hyp-PDT (35); or even negatively regulate ICD in some settings, e.g., autophagy in case of Hyp-PDT (34) (Table 5). Thus, it will be important to expand our molecular knowledge of ICD to as many experimental settings as possible.

**Table 5:** **A list of molecular and immunological components crucial for regulation of ICD**.

Molecular or immunological components	Acting on the level of?	Role in regulating ICD or ICD-related determinants for various therapies/inducers			Confirmed by which experimental intervention?	Reference
		Positive regulation	Negative regulation	No role in regulation		
Actin cytoskeleton	Cancer cells	Anthracyclines, hypericin-PDT	–	–	Pharmacological inhibitors of actin polymerization	(35, 123)
ATG5, ATG7, or BECN1	Cancer cells	Anthracyclines, oxaliplatin	Hypericin-PDT	Newcastle disease virotherapy	ATG5, ATG7 or BECN1 si/shRNA, ATG5 KO MEFs, or transgenic mice model of spontaneous melanoma with *Atg7*^−/−^ phenotype or pharmacological inhibitors of macroautophagy	(34, 43, 112)
BAX/BAK	Cancer cells	Anthracyclines, hypericin-PDT	–	–	BAX/BAK KO MEFs or Bax/Bak si/shRNA	(35, 123)
Calreticulin	Cancer cells	Anthracyclines, radiotherapy, oxaliplatin, hypericin-PDT	–	–	CRT si/shRNA	(35, 41, 116, 123)
Caspase 1	Host immune system	Anthracyclines and/or oxaliplatin	–	–	*Casp1*^−/−^ mice	(36)
Caspase-8	Cancer cells	Anthracyclines	–	Hypericin-PDT	Caspase-8 si/shRNA or HeLa cancer cells expressing CrmA (a caspase-8 inhibitory protein)	(35, 123)
CD4^+^/CD8^+^ T cells	Host immune system	Anthracyclines and/or oxaliplatin, hypericin-PDT, high hydrostatic pressure, bortezomib, vorinostat, photofrin-PDT, Newcastle disease virotherapy, cyclophosphamide	–	–	Antibody-based depletion; *Ex vivo* co-culture experiments	(34, 43, 100, 102, 152, 161, 162, 166, 191)
CXCL10	Host immune system	Anthracyclines and/or oxaliplatin	–	–	Recombinant protein	(102, 141)
CXCR3	Host immune system	Anthracyclines and/or oxaliplatin	–	–	*Cxcr3*^−/−^ mice or antibody-based blockade	(141)
eIF2α-P	Cancer cells	Anthracyclines	–	Hypericin-PDT	MEFs expressing non-phosphorylable version of eIF2α-P, salubrinal or pharmacological inhibitors of GADD34	(35, 123)
ER-Ca^2+^	Cancer cells	Anthracyclines	–	Hypericin-PDT	BAPTA, a Ca^2+^ chelator or Reticulon-1C overexpression;	(35)
ERp57	Cancer cells	Anthracyclines	–	Hypericin-PDT	ERp57 si/shRNA or ERp57 KO MEFs	(35, 116)
ER-to-Golgi transport	Cancer cells	Anthracyclines, hypericin-PDT	–	–	Brefeldin A, a secretory pathway inhibitor	(35, 123)
HMGB1	Cancer cells	Anthracyclines	–	–	HMGB1 si/shRNA	(70)
HSP90	Cancer cells	Bortezomib	–	–	Pharmacological HSP90 inhibitors	(66, 67, 100)
HSP70	Cancer cells	Shikonin	–	–	Antibody-mediated protein depletion	(192)
IFN-α/β or IFN-α-receptor	Cancer cells	Anthracyclines, cyclophosphamide, and/or oxaliplatin	–	–	Antibody-based blockade or recombinant proteins (wherever applicable)	(141, 152)
IFN-γ and IFN-γ-receptor	Host immune system	Anthracyclines and/or oxaliplatin	–	–	*Ifng*^−/−^ or *Ifngr1*^−/−^ mice	(70, 102)
IL17A or IL17A-receptor	Host immune system	Anthracyclines and/or oxaliplatin	–	–	*Il17a*^−/−^ or *Il17ra*^−/−^ mice	(36, 193)
IL1-receptor	Host immune system	Anthracyclines and/or oxaliplatin	–	–	*Il1r1*^−/−^ mice	(36)
IL-1β	Host immune system	Anthracyclines and/or oxaliplatin	–	–	Antibody-based blockade	(36)
Lipid rafts	Cancer cells	Mitoxantrone	–	Hypericin-PDT	MBC, a cholesterol-chelator that disrupts lipid rafts	(35)
LRP1	Cancer cells	Mitoxantrone, hypericin-PDT	–	–	LRP1 shRNA, LRP1 KO MEFs, LRP1 KO CHO cells and LRP1 overexpression in CHO cells	(35)
LY96 and MyD88 (TLR-adaptors)	Host immune system	Anthracyclines and/or oxaliplatin	–	–	*Ly96*^−/−^ or *Myd88*^−/−^ mice	(102)
NLRP3	Host immune system	Anthracyclines and/or oxaliplatin	–	–	*Nlrp3*^−/−^ mice	(36)
P2 × 7 receptor	Host immune system	Anthracyclines and/or oxaliplatin	–	–	*P2rx7*^−/−^ mice	(36)
Perforin	Host immune system	Anthracyclines and/or oxaliplatin	–	–	*Prf1*^−/−^ mice	(36, 70, 102)
PERK	Cancer cells	Anthracyclines, hypericin-PDT, wogonin	–	–	PERK si/shRNA, PERK KO MEFs	(35, 123, 167)
PI3K p110α	Cancer cells	Anthracyclines, hypericin-PDT, wogonin	–	–	PI3K p110α shRNA or wortmannin, a pharmacological inhibitor	(35, 167)
Rag2	Host immune system	Anthracyclines and/or oxaliplatin, vorinostat, cyclophosphamide, photofrin-PDT, Newcastle disease virotherapy	–	–	*Rag2*^−/−^ mice	(43, 70, 102, 152, 161, 162, 166)
STAT3	Cancer cells	Anthracyclines and/or oxaliplatin	–	–	*Stat3*^−/−^ cancer cells	(194)
TLR3	Cancer cells	Anthracyclines and/or oxaliplatin	–	–	TLR3 si/shRNA or *Tlr3*^−/−^ cancer cells	(141)
TLR4	Host immune system	Anthracyclines and/or oxaliplatin	–	–	*Tlr4*^−/−^ mice	(70, 102)
TNF or TNF-receptor	Host immune system	Anthracyclines and/or oxaliplatin	–	–	*Tnf*^−/−^ or *Tnfr1*^−/−^ mice	(102)
LAMP2A	Cancer cells?	Mitoxantrone and hypericin-PDT	–	–	LAMP2A KO MEFs	(112)

*ATG, autophagy-related protein; BECN1, beclin-1; CD, cluster of differentiation; CRT, calreticulin; CXCL, C-X-C ligand; CXCR, C-X-C motif receptor; eIF2, eukaryotic initiation factor 2; ER, endoplasmic reticulum; ERp57, endoplasmic reticulum protein 57; HMGB1, high-mobility group box 1; HSP, heat shock protein; Hyp-PDT, hypericin-based photodynamic therapy; ICD, immunogenic cell death; IFN, interferon; IL, interleukin; KO MEFs, knock-out murine embryonic fibroblasts; LAMP, lysosome-associated membrane glycoprotein; LRP1, low-density lipoprotein receptor-related protein 1; MBC, methyl-β-cyclodextrin; NLRP3, NOD-like receptor family, pyrin domain containing 3; PERK, protein kinase RNA-like endoplasmic reticulum kinase; PI3K, phosphoinositide 3-kinase; PRF, perforin; TLR, toll-like receptor; TNF, tumor necrosis factor*.

## Immunogenic Cell Death from Bench to Bedside

The relevance of ICD has been verified in a number of rodent models, with a variety of chemical and physicochemical ICD inducers (26, 102). Table 6 summarizes the most prominent mouse or rat models used so far for the characterization and study of ICD. For the moment, ICD has been mostly investigated in heterotopic syngeneic subcutaneous models (195). Within such models, inter-species differences (mouse *versus* rats), inter-strain differences (among BALB/c, C57BL/6, C3H and KMF mice), and inter-cell line differences, as well as differences in therapeutic setups (prophylactic *versus* curative) have been amply accounted for (Table 6). Nevertheless, there is predominance in the use of cancer cells derived from carcinogen-induced tumors and transplanted subcutaneously (Table 6). In very few cases, ICD has been characterized in either orthotopic (for NDV) or spontaneous (for anthracyclines) tumor murine models (Table 6). This has been questioned as a prominent Achilles’ heel of ICD research (195). While this criticism is valid, it has to be recognized that no rodent model is perfect at all immunological levels (196).

**Table 6:** **A list of prominent preclinical mice or rat models used for analysis of ICD**.

ICD inducer	Mice tumor models utilized for positive ICD characterization or ICD “restoration/rescue” analysis			
	Heterotopic subcutaneous mice or rat models	Orthotopic mice models	Spontaneous tumor mice models	Carcinogen-induced tumor models
Anthracyclines	CT26 cells in BALB/c mice – prophylactic immunization model (41, 70, 111, 123, 197) and curative tumor model (41, 70, 111, 197); MCA205 cells in C57BL/6 mice – prophylactic immunization and curative tumor model (36, 70, 111, 130); MCA-2/-4 cells in C57BL/6 mice – curative tumor model (36); D122 cells in C57BL/6 mice – prophylactic immunization model (145); AY27 cells in Fischer 344 rats – prophylactic immunization model (42)	–	MMTV-*Neu*T breast cancer mice model – curative set-up (198); *Braf*^Ca/+^; *Pten*^fl/fl^-melanoma mice model – curative set-up (199)	–
Anti-EGFR antibody (7A7)	D122 cells in C57BL/6 mice – curative tumor model and prophylactic immunization model (145)	–	–	–
Bleomycin	CT26 cells in BALB/c mice – curative tumor model (146)	–	–	–
Bortezomib	67NR cells in BALB/c mice – prophylactic immunization model with use of stimulated DCs (200); B16 cells in C57BL/6 mice – curative tumor model, combination treatment with AdVMART1/DC and bortezomib is significantly better than bortezomib alone (201); HM-1 cells in C57BL/6 x C3/He F_1_ origin mice – prophylactic immunization model (202)	–	–	–
CD40L-encoding Oncolytic Adenovirus	MB49 cells in C57BL/6 mice – curative tumor model (147)	–	–	–
*Clostridium difficile* toxin B	CT26 cells in BALB/c mice – prophylactic immunization model (149)	–	–	–
Coxsackievirus B3	A549 and EBC-1 cells in nude BALB/c mice – curative tumor model (150)	–	–	–
Cyclophosphamide	EG7 cells in C57BL/6 mice (152); AB1-HA cells in BALB/c mice – curative tumor model followed by resistance to challenge with live cells (203)	–	–	–
Hypericin-based PDT	CT26 cells in BALB/c mice – prophylactic immunization model (35); – curative tumor model (184); AY27 cells in Fischer 344 rats – prophylactic immunization model (42); B78 cells in C57BL/6 mice – prophylactic immunization model (30)	–	–	–
Microwave thermal ablation	K7M2 cells in BALB/c mice or UMR106 cells in SD rats – prophylactic immunization model (158)	–	–	–
Newcastle disease virus (NDV)	B16 cells in C57BL/6 mice – curative tumor model (159)	GL261 cells in C57BL/6 mice – curative tumor model (43)	–	–
Oxaliplatin	CT26 cells in BALB/c mice – prophylactic immunization model (123, 197); – curative tumor model (197); EL4 cells in C57BL/6 mice – curative tumor model (36); EG7 cells in C57BL/6 mice – curative tumor model (36); EG7 cells in C3H mice – prophylactic immunization model (70)	–	–	–
Photofrin-based PDT	EMT6 cells in BALB/c mice – curative tumor model (161); SCCVII cells in C3H/HeN mice – curative tumor model (162, 163)	–	–	–
Radiotherapy	CT26 cells in BALB/c – prophylactic immunization model (204); 410.4 cells in BALB/c mice – prophylactic immunization model (205); EG7 cells in C57BL/6 mice and SCC VII cells in C3H mice – prophylactic immunization model (206); B16F10 cells in C57BL/6 mice – prophylactic immunization model with the use of irradiated cancer cells, as well as DCs stimulated with irradiated cancer cells (207)	–	–	–
RIG-I-like helicases (RLH) ligand	Panc02 cells in C57BL/6 mice – prophylactic immunization and curative tumor model (165)	–	–	–
Septacidin	MCA205 cells in BALB/c mice – prophylactic set-up (139);	–	–	–
Shikonin	B16 cells in C57BL/6 mice – prophylactic immunization model (160); P388 cells in KMF mice – curative tumor model (208)	4T1 cells in BALB/c mice – curative tumor model (192);	–	–
UVC irradiation	CT26 cells in BALB/c mice – prophylactic immunization model (204); EG7 cells in C57BL/6 mice – curative tumor model (152)	–	–	–
Vorinostat	MC38 or Eμ-myc 4242/299 lymphoma in C57BL/6 mice – curative tumor set-up (166)	–	–	–
High hydrostatic pressure	No mice or rat based preclinical data available to support their ICD-functions			
Pt^II^ N-heterocyclic carbene complex				

*DC, dendritic cell; ICD, immunogenic cell death; PDT, photodynamic therapy*.

As a recent systematic review summarized (196), heterotopic murine models suffer from a number of caveats, including the inability to recapitulate the early interaction between transformed cells and the immune system and the incompatibility between the cancer type and the site-of-transplantation (196). Orthotopic murine models are useful as they overcome the cancer cell-tissue type incompatibility issue (196). While genetically engineered tumor murine models (GEMMs) overcome most of the issues mentioned above, they come with their own set of shortcomings, including a limited genetic mosaicism, a low tumor heterogeneity, a lack of well-defined immunogenic TAAs, the presence of unintended “passenger” genetic modifications, and a reduced mutational spectrum (196). Many of these parameters are critical for responses to immunotherapy/ICD. For instance, the lack of well-defined immunogenic TAAs was the reason why preliminary results obtained in spontaneously developing murine tumors disputed the very existence of TAAs (11). Similarly, a high mutational spectrum (which produces considerable amounts of neo-antigens) has been found to be mandatory for the clinical efficacy of checkpoint blockers (209). Last (but not least), laboratory rodent models in general are associated with some critical issues, including the fact that a high level of inbreeding (which produces a number of shortcomings e.g., homozygous recessive defects) reduces the general immunological fitness, responsiveness and diversity in these models (196, 210, 211). Moreover, numerous immunological differences between mouse and humans tend to affect the translational relevance of the findings obtained (26, 211, 212). Also, the time frames of tumor growth rates between rodent models and humans are relatively divergent (196, 213, 214). This further complicates clinical translation of immunotherapeutic paradigms since the level of immunosurveillance and immunoediting experienced by human tumors can be much higher than any rodent tumor model.

In summary, it would be ideal to test ICD across as many different rodent models as possible, in order to determine the features that can be exploited for therapeutic purposes in humans. Moreover, if ICD fails in a specific experimental model, active effort should be made to characterize the mechanisms behind such failure, since resistance phenotypes can have profound clinical implications. This emerges from various studies summarized in Table 7. Indeed, several ICD resistance mechanisms exist operating at both the cancer cell and the immune system level, which have been characterized in different experimental models. Several of these resistance mechanisms have also been identified in cancer patients, thereby justifying further studies along these lines Table 7.

**Table 7:** **Existence of intrinsic or naturally occurring resistance to ICD in experimental cancer models**.

ICD inducer(s)	Experimental set-up where resistance was observed	Reason behind resistance	Rescued by?	Clinical applicability verified?	Reference
***In vivo* preclinical setting (cancer cell or host immune system-level resistance)**					
Anthracyclines or anthracycline plus oxaliplatin	C3H mice with naturally occurring *tlr4* mutation	Host immune system-level resistance: defective *TLR4* in C3H mice causes failure of HMGB1-mediated immunity thereby leading to resistance to anti-cancer vaccination effect associated with anthracyclines treatment	Adoptive transfer of TLR4-expressing DCs loaded with dying tumor cells	Yes; breast cancer, colon cancer, and lung cancer patients carrying TLR4 gene mutation that ablates its ability to bind its ligands is associated with worse prognosis post-treatment	(215)
Doxorubicin	AT-3 or 4T1.2 breast cancer cells in C57BL/6 or BALB/c mice, respectively	Cancer cell-level resistance: CD73 overexpression confers chemo-resistance to doxorubicin by suppressing anti-tumor immunity through A2A adenosine receptors	Blockade of CD73	Yes; in triple-negative breast cancer patients, high CD73 in anthracycline-treatment set-up associated with lower rate of complete responses	(216)
Mitoxantrone and Hypericin-PDT	AY27 rat bladder cancer cells in Fischer 344 rats	Cancer cell-level resistance: low endogenous CRT levels, resulted in severely reduced surface-CRT upon treatment with mitoxantrone or Hyp-PDT; this in turn compromised immunogenic phagocytic clearance and anti-cancer vaccination effect	Exogenous addition of recombinant CRT	Yes; high tumoral *CALR* levels correlated with high expression of phagocytosis-associated genes and predicted for prolonged survival after RT or PTX treatment of lung or ovarian cancer patients respectively	(42)
Oxaliplatin	Autochthonous transgenic adenocarcinoma of the mouse prostate (TRAMP) model of metastatic prostate cancer	Host immune system-level resistance: immunosuppressive B cells expressing IgA, IL10 and PD-L1 cause resistance to anti-tumorigenic effects of oxaliplatin	Genetic or pharmacological depletion of B cells	Not directly, but possible validity is supported by human patient data showing that IL-10 expressing IgA+ cells are abundant in therapy-resistant prostate cancer and are negative prognostic indicators	(217)
***In vitro* preclinical setting (cancer cell-level resistance)**					
Anthracycline	SH-SY5Y neuroblastoma cell line	Anthracycline treatment of these cells failed to induce surface-CRT due to reduced capacity to efflux ER-Ca^2+^ into cytosol	Overexpression of reticulon-1C	–	(132)
Doxorubicin	HT29-dx and HT29 iNOS-cells (human colon cancer cells)	Doxorubicin failed to induce NO synthesis, which resulted in reduced toxicity, reduced surface-CRT and subsequently compromised immunogenic phagocytic clearance and DC stimulation	Addition of sodium nitroprusside or a NO donor	–	(218)
Doxorubicin	MDR+ human cancer cells (HT29-dx, A549-dx and MCF-7-dx)	Increased MDR levels caused increased P-glycoprotein expression which caused resistance to doxorubicin-induced ICD by affecting immunogenic phagocytic removal	Addition of zoledronic acid	Not directly	(219)

*CD, cluster of differentiation; CRT or *CALR*, calreticulin; DC, dendritic cells; ER, endoplasmic reticulum; HMGB1, high-mobility group box-1 protein; HSP, heat shock protein; Hyp-PDT, hypericin-photodynamic therapy; ICD, immunogenic cell death; IL, interleukin; MDR, multiple drug-resistance; NO, nitric oxide; NOS, nitric oxide synthase; PD-L1, programed cell death protein ligand 1; PTX, paclitaxel; RT, radiotherapy; TLR, toll-like receptor*.

A considerable amounts of clinical findings support the relevance of ICD or ICD-related signatures in (at least subsets of) cancer patients. As summarized in Table 8, various ICD-linked (specific) parameters have been associated with the prognosis of cancer patients treated with clinically relevant ICD inducers (like anthracyclines, oxaliplatin, paclitaxel, or radiotherapy). Moreover, it is becoming clear that ICD-related or ICD-derived (immunological) genetic signatures (e.g., a *MX1*-centered metagene, a *CXCR3*-*PRF1*-*CASP1*-centered metagene, an *ASAH1*-centered metagene) can be positively associated with good prognosis in patients affected by various neoplasms, including breast, lung, and ovarian malignancies (141, 188, 220). These observations indicate that ICD or ICD-relevant parameters may have prognostic or predictive relevance in at least a subset of cancer patients. It will be important to characterize new and more specific ICD-associated parameters linked to patient prognosis as well as biomarkers that may predict improved disease outcome in cancer patient treated with ICD inducers. Of note, considering the current clinical experience with immunotherapies (209, 221), the patients with an increased likelihood to benefit from ICD inducers are probably those that display pre-existing (baseline) immune reactivity against cancer cells (220, 222, 223). This may depend on the ability of ICD to reboot and/or revive pre-existing TAA-directed immunity rather to prime *de novo* immune reactivity (5, 191, 224). In future, it would be crucial to characterize biomarkers that allow clinicians to delineate patients with reduced baseline immune reactivity against malignant cells so that proper combinatorial therapies involving ICD inducers can be implemented.

**Table 8:** **A list of clinical observations supporting the existence of ICD in cancer patients**.

ICD inducer	Standard-of-care therapy or regularly applied palliative therapy in clinic?	ICD-related characteristics regulating clinical patient prognosis or treatment-responsiveness
Anthracyclines	Yes	*P2RX7* loss-of-function mutation that compromises ICD also negatively affects MFS in breast cancer patients treated with adjuvant anthracyclines (36); breast cancer patients possessing a wild-type *TLR4* benefited more from the anthracyclines than those who possessed a mutated *TLR4* that compromises ICD (70); an *MX1*-centered Type I IFN signature in anthracycline-treated breast cancer patients predicts for improved disease outcome (141); combined positivity for cytoplasmic LC3B+ puncta and nuclear HMGB1 is a positive predictor of improved survival following adjuvant anthracycline-based chemotherapy (225)
High hydrostatic pressure	No; but HHP-based anticancer DC vaccines are currently being applied in clinical trials against prostate cancer and ovarian cancer (155)	No data are available
Hypericin-based PDT	No; but few clinical trials have been carried out for non-melanoma skin cancer (226), cutaneous T-cell lymphoma (227), mesothelioma (228), and basal or squamous cell carcinoma (229)	No data are available
Oncolytic adenoviruses	No; but oncolytic adenoviruses are currently being applied in various clinical trials in cancer patients	Serum HMGB1 levels and the temporal change in their levels during treatment was identified as a prognostic and predictive biomarker in cancer patients (230)
Oxaliplatin	Yes	Similar to anthracyclines, cancer patients possessing wild-type *TLR4* exhibited prolonged PFS and OS in comparison to patients bearing the loss-of-function allele of *TLR4* (197)
Paclitaxel	Yes	High tumoral *CALR* levels in paclitaxel-treated ovarian cancer patients associated with prolonged OS/PFS as well as increased expression levels of various phagocytosis-associated genes (42)
Photofrin-based PDT	Yes; FDA-approved for application in esophageal and lung cancer (231)	No data available
Radiotherapy	Yes	In patients of eosophageal squamous cell carcinoma (ESCC) receiving chemo-radiotherapy significant increase in serum HMGB1-levels and increased intra-tumoral staining of HMGB1 correlated with better patient survival (232); high tumoral *CALR* levels in radiotherapy-treated lung cancer patients associated with prolonged OS as well as increased expression levels of various phagocytosis-associated genes (42)
Shikonin	No; but shikonin is currently being applied in an observational clinical study of breast cancer patients (NCT01287468)	No data are available
UVC irradiation	No; but UV treatment is sometimes applied for the preparation of clinical cell-based anticancer vaccines (233)	No data are available
Bortezomib, Anti-EGFR antibody (7A7), bleomycin, cyclophosphamide, microwave thermal ablation, vorinostat	Yes	No data are available
Coxsackievirus B3; *Clostridium difficile* toxin B; Microwave thermal ablation; Newcastle disease virus (NDV); RIG-I-like helicases (RLH) ligand; Septacidin; Pt^II^ N-heterocyclic carbene complex; Patupilone	No	No data are available

*CRT or *CALR*, calreticulin; HMGB1, high-mobility group box-1 protein; Hyp-PDT, hypericin-photodynamic therapy; ICD, immunogenic cell death; IFN, interferon; OS, overall survival; PFS, progression-free survival; TLR, toll-like receptor*.

## Confronting the Clinical Realities of Anti-Tumor Immunity

**Figure 2:**
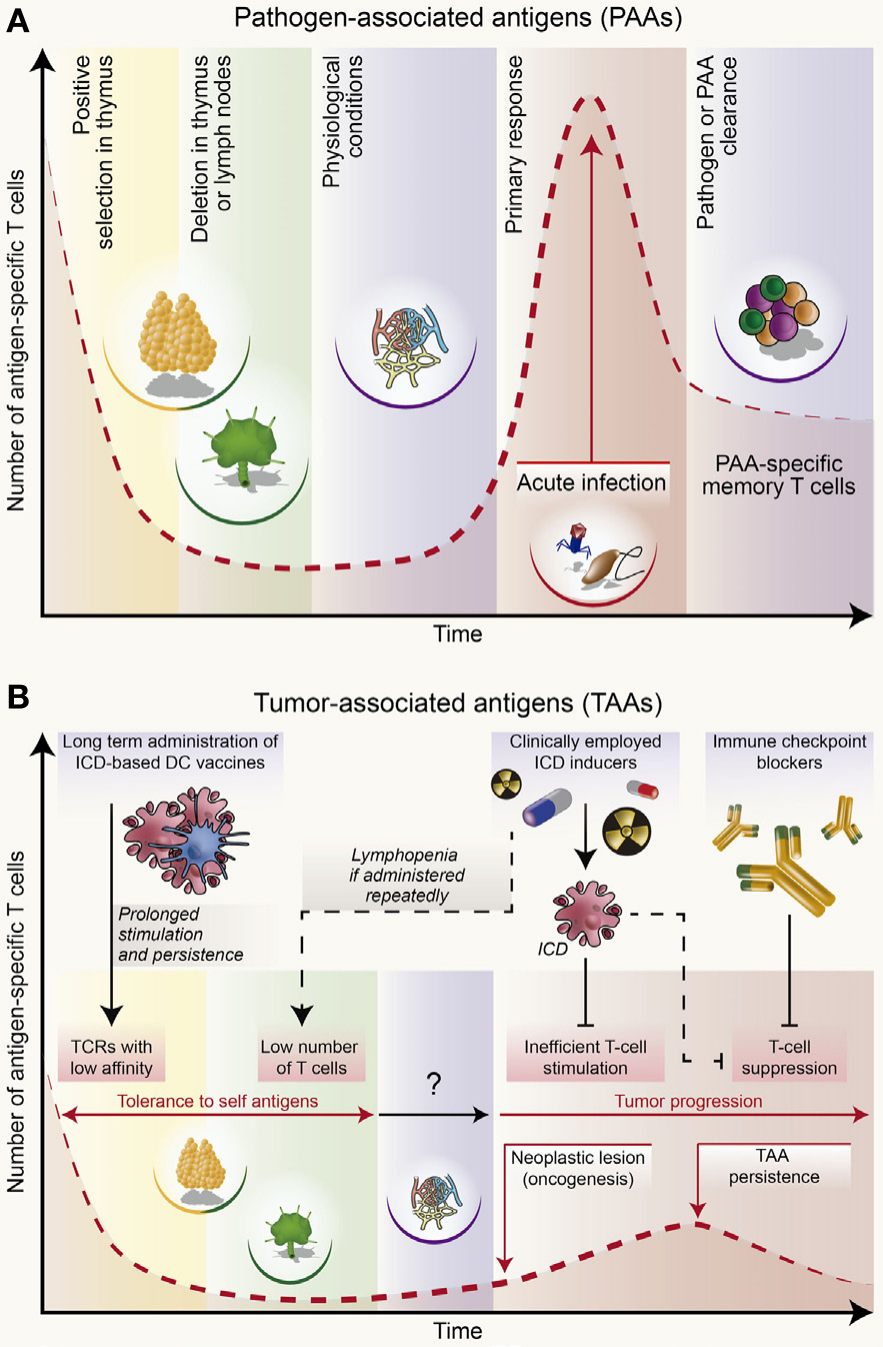
**Population dynamics of antigen-specific T cells during an immune response to infection or cancer**. **(A)** T cells capable of putatively recognizing non-self, pathogen-associated antigens (PAAs) are not exposed to negative selection in the thymus or peripheral organs like lymph nodes. This allows for the constitutive presence of T lymphocytes bearing high-affinity T-cell receptor (TCR) in naïve conditions. Upon infection, these cells undergo robust expansion and acquire potent effector functions, hence driving an immune response that clears the pathogen and PAAs. Finally, PAA-specific T cells undergo contraction along with the establishment of immunological memory. To a limited extent, T cells reacting against PAAs expressed by virus-induced tumors may exhibit similar (although not identical) responses. **(B)** T cells that may recognize self or close-to-self antigens expressed by virus-unrelated malignancies undergo robust negative selection in the thymus and lymph nodes. Thus, all putative T lymphocytes bearing a high-affinity TCR against tumor-associated antigens (TAAs) are eliminated. However, some leakiness in this process allows for the persistence of TAA-specific T lymphocytes with low-affinity TCR, although at very low precursor frequencies. This is one of the reasons why in some individuals immunosurveillance at some stage fails to impede tumor progression. As malignant lesions progress, the amount of TAAs increases, causing a weak rise in TAA-specific T cells. However, tumor progression is generally coupled with the establishment of robust immunosuppressive networks that potently inhibit such TAA-targeting T cells. In this context, the administration of immunogenic cell death (ICD) according to a schedule that does not lead to lymphodepletion can favor the stimulation of TAA-targeting T cells and (re)instate immunosurveillance. Combining ICD inducers with checkpoint-blocking agents may further boost TAA-targeting immune responses. However, these treatments may not ensure the lifelong persistence of TAA-recognizing T cells, some of which are susceptible to elimination through tolerance mechanisms. Anticancer vaccines may counteract, at least to some extent, such loss. The figure was partly inspired from Baitsch et al. (234).

It is well-established that the response of cancer patients to immunotherapy relies on the activity of effector T cells [that employ their T-cell receptors (TCRs) for recognizing TAAs]. However, these TAA-targeting T cells may also constitute obstacles for effective anti-tumor immunity (234). As opposed to T lymphocytes recognizing pathogen-associated antigens (PAAs) (Figure 2), indeed, T cells directed against some TAAs (derived from non-mutated proteins that are source of self or near-to-self antigens) are developmentally subjected to negative selection in the thymus and peripheral lymphoid organs (234, 235) (Figure 2). As a result, T cells bearing TCRs with high affinity for self antigens (including some TAAs) are clonally deleted to avoid auto-immunity (234–237) (Figure 2). However, some “leakiness” in this process allows TAA-specific T cells possessing TCRs with low affinity to escape deletion (234, 236, 237) and persist, although at low precursor frequencies (238) (Figure 2). Unfortunately, as compared to PAA-specific T cells, which bear high-affinity TCRs (Figure 2), TAA-specific T cells exhibit limited effector and memory functions (234, 239). Coupled with the tendency of progressing tumors to generate a highly immunosuppressive microenvironment, this renders the insurgence of lifelong protective immunity nearly impossible (234). Of note, central and peripheral tolerance may not affect T cells reactive toward neo-tumor-specific antigens (neo-TSAs) e.g., tumor-specific neo-antigens that are generated *de novo* in the course of tumor progression because of mutational events (240, 241). However, the extent to which such neo-TSAs can elicit consistent “immunodominant” T cell reactivity is still a matter of investigation (240, 241). Nevertheless, in this context, inefficient T-cell stimulation can be overcome through the ICD-based improvement of effector T-cell functions (102). ICD can be further combined with checkpoint-blocking therapies, which potently reverse immunosuppression (209, 242). However, the lifelong maintenance of anti-tumor T cells remains a particularly hard challenge.

In the clinical reality, anticancer agents are administered to patients in a limited number of cycles. Even if these therapeutic regimens may attain optimal efficacy in terms of ICD induction, they are unlikely to ensure the lifelong persistence of TAA-directed T cells with low-affinity TCR (234, 243). This probably reflects the contraction of TAA-targeting T cells occurring once the immunostimulatory stimulus provided by ICD ceases, owing to peripheral tolerance mechanisms (234). Clinically, it may not be feasible to administer ICD inducers repeatedly over time, since many of them can cause lymphopenia (which negatively affects disease outcome), or are associated with other side effects (244). It has been proposed that active immunization with ICD-based anticancer vaccines (which are associated with robust immunogenicity) given in a repetitive manner may achieve this goal (Figure 2) (234, 243, 245). Thus, it will be important to test whether the long-term administration of ICD-based anticancer vaccines can sustain the effector function of TAA-specific T cells bearing low-affinity TCRs, hence, ensuring lifelong disease-free survival. Of note, in the case of hematological malignancies, this issue could be overcome upon the adoptive transfer of CTLs expressing chimeric antigen receptors (CARs) (1). However, whether CAR-expressing CTLs generate protective immunological memory in the absence of considerable side effects remains to be determined. Moreover, the use of this therapeutic strategy against solid malignancies is relatively challenging owing to lack of well-defined “unique” TAAs (1, 246).

## Conclusion

The model of ICD has been considerably refined since the initial identification of a cell death modality manifesting apoptotic features but able to induce an adaptive immune response. This model strives to integrate several phenomena observed throughout the second half of the twentieth century in one therapeutically relevant platform. However, as discussed above, several challenges still need to be addressed. First, comprehensive testing should be performed in advanced experimental settings like GEMMs or orthotopic tumor models. Second, ICD resistance mechanisms should be characterized with precision. Third, various issues linked to the successful translation of ICD to cancer therapy will have to be resolved, including (but not limited to) treatment schedules, dosages, and combinatorial strategies. This translational drive also needs to be coupled with effective strategies for the discovery of new and effective ICD inducers. Drug screening programs are often complicated by the possibility of false-positive (due to bystander presence of DAMPs) (30) or false-negative (due to limited number of biomarkers used for screening) hits. This issue can only be ironed out by discovering new and common regulators of ICD, and integrating them into existing screening platforms. Last, but not least, it will be important to identify new ICD-related/derived biomarkers that can be used to improve current protocols of patient stratification and clinical decision making. We are positive that all these objectives are at reach.

## Author Contributions

ADG did the literature study, data collection, as well as conceived and wrote the manuscript. PA provided senior supervision and guidance, conceived the paper, helped in writing, and critically revised the manuscript. LG improved and edited the manuscript. JMBSP helped with the preparation of figures. All authors participated in the critical reading of the manuscript (wherever applicable), approved content and conclusions, as well as helped in ensuring the accuracy of cited literature.

## Conflict of Interest Statement

Akseli Hemminki is shareholder in Targovax AG and TILT Biotherapeutics Ltd. The remaining authors have no conflict of interest to declare.

## Abbreviations

DAMP, damage-associated molecular pattern; DC, dendritic cell; ER, endoplasmic reticulum; GEMM, genetically engineered murine model; HSP, heat shock protein; Hyp, hypericin; ICD, immunogenic cell death; NDV, Newcastle disease virotherapy; PDT, photodynamic therapy; ROS, reactive oxygen species.

## References

[1] Galluzzi L Vacchelli E Bravo-San Pedro JM Buque A Senovilla L Baracco EE Classification of current anticancer immunotherapies. Oncotarget (2014) 5(24):12472–508. 10.18632/oncotarget.2998 25537519 PMC4350348

[2] Kepp O Tesniere A Zitvogel L Kroemer G. The immunogenicity of tumor cell death. Curr Opin Oncol (2009) 21(1):71–6. 10.1097/CCO.0b013e32831bc375 19125021

[3] Garg AD Dudek AM Agostinis P. Cancer immunogenicity, danger signals, and DAMPs: what, when, and how? Biofactors (2013) 39(4):355–67. 10.1002/biof.1125 23900966

[4] Blankenstein T Coulie PG Gilboa E Jaffee EM. The determinants of tumour immunogenicity. Nat Rev Cancer (2012) 12(4):307–13. 10.1038/nrc3246 22378190 PMC3552609

[5] Garg AD Nowis D Golab J Vandenabeele P Krysko DV Agostinis P. Immunogenic cell death, DAMPs and anticancer therapeutics: an emerging amalgamation. Biochim Biophys Acta (2010) 1805(1):53–71. 10.1016/j.bbcan.2009.08.003 19720113

[6] Anguille S Smits EL Lion E van Tendeloo VF Berneman ZN. Clinical use of dendritic cells for cancer therapy. Lancet Oncol (2014) 15(7):e257–67. 10.1016/S1470-2045(13)70585-0 24872109

[7] Chiang CL Kandalaft LE Coukos G. Adjuvants for enhancing the immunogenicity of whole tumor cell vaccines. Int Rev Immunol (2011) 30(2–3):150–82. 10.3109/08830185.2011.572210 21557641

[8] Parish CR. Cancer immunotherapy: the past, the present and the future. Immunol Cell Biol (2003) 81(2):106–13. 10.1046/j.0818-9641.2003.01151.x 12631233

[9] Coley WB The treatment of malignant tumors by repeated inoculations of erysipelas: with a report of ten original cases. Am J Med Sci (1893) 105:487–511. 10.1097/00000441-189305000-00001 1984929

[10] Tsung K Norton JA. Lessons from Coley’s toxin. Surg Oncol (2006) 15(1):25–8. 10.1016/j.suronc.2006.05.002 16814541

[11] Coulie PG Van den Eynde BJ van der Bruggen P Boon T. Tumour antigens recognized by T lymphocytes: at the core of cancer immunotherapy. Nat Rev Cancer (2014) 14(2):135–46. 10.1038/nrc3670 24457417

[12] Liao SK Carr DH Comparative immunogenicity of irradiated, neuraminidase treated, and fused cells of a strain-restricted sarcoma. Z Krebsforsch klin Onkol Cancer Res Clin Oncol (1974) 82(2):133–42. 4370664 10.1007/BF00284498

[13] Milas L Withers HR. Nonspecific immunotherapy of malignant tumors. Radiology (1976) 118(1):211–8. 10.1148/118.1.211 1105663

[14] Bogden AE Esber HJ. Influence of surgery, irradiation, chemotherapy, and immunotherapy on growth of a metastasizing rat mammary adenocarcinoma. Natl Cancer Inst Monogr (1978) (49):97–100. 748802

[15] Dickson JA Shah SA. Hyperthermia: the immune response and tumor metastasis. Natl Cancer Inst Monogr (1982) 61:183–92. 7177177

[16] Suit HD Walker AM. Assessment of the response of tumours to radiation: clinical and experimental studies. Br J Cancer Suppl (1980) 4:1–10. 7000117 PMC2149218

[17] Richert L Or A Shinitzky M. Promotion of tumor antigenicity in EL-4 leukemia cells by hydrostatic pressure. Cancer Immunol Immunother (1986) 22(2):119–24. 10.1007/BF00199125 2424597 PMC11038643

[18] Galluzzi L Senovilla L Zitvogel L Kroemer G. The secret ally: immunostimulation by anticancer drugs. Nat Rev Drug Discov (2012) 11(3):215–33. 10.1038/nrd3626 22301798

[19] Green DR Ferguson T Zitvogel L Kroemer G. Immunogenic and tolerogenic cell death. Nat Rev Immunol (2009) 9(5):353–63. 10.1038/nri2545 19365408 PMC2818721

[20] Matzinger P. Tolerance, danger, and the extended family. Annu Rev Immunol (1994) 12:991–1045. 10.1146/annurev.iy.12.040194.005015 8011301

[21] Matzinger P. The danger model: a renewed sense of self. Science (2002) 296(5566):301–5. 10.1126/science.1071059 11951032

[22] Land W Schneeberger H Schleibner S Illner WD Abendroth D Rutili G The beneficial effect of human recombinant superoxide dismutase on acute and chronic rejection events in recipients of cadaveric renal transplants. Transplantation (1994) 57(2):211–7. 10.1097/00007890-199401001-00010 8310510

[23] Seong SY Matzinger P Hydrophobicity: an ancient damage-associated molecular pattern that initiates innate immune responses. Nat Rev Immunol (2004) 4(6):469–78. 10.1038/nri1372 15173835

[24] Rubartelli A Lotze MT. Inside, outside, upside down: damage-associated molecular-pattern molecules (DAMPs) and redox. Trends Immunol (2007) 28(10):429–36. 10.1016/j.it.2007.08.004 17845865

[25] Li G Tang D Lotze MT. Menage a trois in stress: DAMPs, redox and autophagy. Semin Cancer Biol (2013) 23(5):380–90. 10.1016/j.semcancer.2013.08.002 23994764 PMC4085481

[26] Garg AD Martin S Golab J Agostinis P. Danger signalling during cancer cell death: origins, plasticity and regulation. Cell Death Differ (2014) 21(1):26–38. 10.1038/cdd.2013.48 23686135 PMC3858605

[27] Bianchi ME. DAMPs, PAMPs and alarmins: all we need to know about danger. J Leukoc Biol (2007) 81(1):1–5. 10.1189/jlb.0306164 17032697

[28] Bondanza A Zimmermann VS Rovere-Querini P Turnay J Dumitriu IE Stach CM Inhibition of phosphatidylserine recognition heightens the immunogenicity of irradiated lymphoma cells in vivo. J Exp Med (2004) 200(9):1157–65. 10.1084/jem.20040327 15504819 PMC2211859

[29] Stach CM Turnay X Voll RE Kern PM Kolowos W Beyer TD Treatment with annexin V increases immunogenicity of apoptotic human T-cells in Balb/c mice. Cell Death Differ (2000) 7(10):911–5. 10.1038/sj.cdd.4400715 11279536

[30] Dudek-Peric AM Ferreira GB Muchowicz A Wouters J Prada N Martin S Antitumor immunity triggered by melphalan is potentiated by melanoma cell surface-associated calreticulin. Cancer Res (2015) 75(8):1603–14. 10.1158/0008-5472.CAN-14-2089 25762540

[31] Rondas D Crevecoeur I D’Hertog W Ferreira GB Staes A Garg AD Citrullinated glucose-regulated protein 78 is an autoantigen in type 1 diabetes. Diabetes (2015) 64(2):573–86. 10.2337/db14-0621 25204978

[32] Venereau E Casalgrandi M Schiraldi M Antoine DJ Cattaneo A De Marchis F Mutually exclusive redox forms of HMGB1 promote cell recruitment or proinflammatory cytokine release. J Exp Med (2012) 209(9):1519–28. 10.1084/jem.20120189 22869893 PMC3428943

[33] Weyd H Abeler-Dorner L Linke B Mahr A Jahndel V Pfrang S Annexin A1 on the surface of early apoptotic cells suppresses CD8+ T cell immunity. PLoS One (2013) 8(4):e62449. 10.1371/journal.pone.0062449 23638088 PMC3640057

[34] Garg AD Dudek AM Ferreira GB Verfaillie T Vandenabeele P Krysko DV ROS-induced autophagy in cancer cells assists in evasion from determinants of immunogenic cell death. Autophagy (2013) 9(9):1292–307. 10.4161/auto.25399 23800749

[35] Garg AD Krysko DV Verfaillie T Kaczmarek A Ferreira GB Marysael T A novel pathway combining calreticulin exposure and ATP secretion in immunogenic cancer cell death. EMBO J (2012) 31(5):1062–79. 10.1038/emboj.2011.497 22252128 PMC3298003

[36] Ghiringhelli F Apetoh L Tesniere A Aymeric L Ma Y Ortiz C Activation of the NLRP3 inflammasome in dendritic cells induces IL-1beta-dependent adaptive immunity against tumors. Nat Med (2009) 15(10):1170–8. 10.1038/nm.2028 19767732

[37] Elliott MR Chekeni FB Trampont PC Lazarowski ER Kadl A Walk SF Nucleotides released by apoptotic cells act as a find-me signal to promote phagocytic clearance. Nature (2009) 461(7261):282–6. 10.1038/nature08296 19741708 PMC2851546

[38] Iwata A Morgan-Stevenson V Schwartz B Liu L Tupper J Zhu X Extracellular BCL2 proteins are danger-associated molecular patterns that reduce tissue damage in murine models of ischemia-reperfusion injury. PLoS One (2010) 5(2):e9103. 10.1371/journal.pone.0009103 20161703 PMC2816997

[39] Babelova A Moreth K Tsalastra-Greul W Zeng-Brouwers J Eickelberg O Young MF Biglycan, a danger signal that activates the NLRP3 inflammasome via toll-like and P2X receptors. J Biol Chem (2009) 284(36):24035–48. 10.1074/jbc.M109.014266 19605353 PMC2781998

[40] Schaefer L. Extracellular matrix molecules: endogenous danger signals as new drug targets in kidney diseases. Curr Opin Pharmacol (2010) 10(2):185–90. 10.1016/j.coph.2009.11.007 20045380

[41] Obeid M Tesniere A Ghiringhelli F Fimia GM Apetoh L Perfettini JL Calreticulin exposure dictates the immunogenicity of cancer cell death. Nat Med (2007) 13(1):54–61. 10.1038/nm1523 17187072

[42] Garg AD Elsen S Krysko DV Vandenabeele P de Witte P Agostinis P. Resistance to anticancer vaccination effect is controlled by a cancer cell-autonomous phenotype that disrupts immunogenic phagocytic removal. Oncotarget (2015) 6(29):26841–60. 10.18632/oncotarget.4754 26314964 PMC4694957

[43] Koks CA Garg AD Ehrhardt M Riva M Vandenberk L Boon L Newcastle disease virotherapy induces long-term survival and tumor-specific immune memory in orthotopic glioma through the induction of immunogenic cell death. Int J Cancer (2015) 136(5):E313–25. 10.1002/ijc.29202 25208916

[44] Gardai SJ McPhillips KA Frasch SC Janssen WJ Starefeldt A Murphy-Ullrich JE Cell-surface calreticulin initiates clearance of viable or apoptotic cells through trans-activation of LRP on the phagocyte. Cell (2005) 123(2):321–34. 10.1016/j.cell.2005.08.032 16239148

[45] Garcia Fernandez M Troiano L Moretti L Nasi M Pinti M Salvioli S Early changes in intramitochondrial cardiolipin distribution during apoptosis. Cell Growth Differ (2002) 13(9):449–55. 12354754

[46] Sorice M Circella A Cristea IM Garofalo T Di Renzo L Alessandri C Cardiolipin and its metabolites move from mitochondria to other cellular membranes during death receptor-mediated apoptosis. Cell Death Differ (2004) 11(10):1133–45. 10.1038/sj.cdd.4401457 15181455

[47] Korbelik M Banath J Sun J Canals D Hannun YA Separovic D. Ceramide and sphingosine-1-phosphate act as photodynamic therapy-elicited damage-associated molecular patterns: cell surface exposure. Int Immunopharmacol (2014) 20(2):359–65. 10.1016/j.intimp.2014.03.016 24713544 PMC4043304

[48] Horino K Nishiura H Ohsako T Shibuya Y Hiraoka T Kitamura N A monocyte chemotactic factor, S19 ribosomal protein dimer, in phagocytic clearance of apoptotic cells. Lab Invest (1998) 78(5):603–17. 9605185

[49] Nishimura T Horino K Nishiura H Shibuya Y Hiraoka T Tanase S Apoptotic cells of an epithelial cell line, AsPC-1, release monocyte chemotactic S19 ribosomal protein dimer. J Biochem (2001) 129(3):445–54. 10.1093/oxfordjournals.jbchem.a002876 11226885

[50] Peter C Wesselborg S Lauber K Role of attraction and danger signals in the uptake of apoptotic and necrotic cells and its immunological outcome. In: Krysko DV Vandenabeele P, editors. Phagocytosis of Dying Cells. Berlin: Springer Science + Business Media B.V. (2009). p. 63–101.

[51] Yamamoto T. Roles of the ribosomal protein S19 dimer and the C5a receptor in pathophysiological functions of phagocytic leukocytes. Pathol Int (2007) 57(1):1–11. 10.1111/j.1440-1827.2007.02049.x 17199736

[52] Struck J Uhlein M Morgenthaler NG Furst W Hoflich C Bahrami S Release of the mitochondrial enzyme carbamoyl phosphate synthase under septic conditions. Shock (2005) 23(6):533–8. 15897806

[53] Krysko DV Garg AD Kaczmarek A Krysko O Agostinis P Vandenabeele P. Immunogenic cell death and DAMPs in cancer therapy. Nat Rev Cancer (2012) 12(12):860–75. 10.1038/nrc3380 23151605

[54] Pullerits R Bokarewa M Jonsson IM Verdrengh M Tarkowski A. Extracellular cytochrome c, a mitochondrial apoptosis-related protein, induces arthritis. Rheumatology (Oxford) (2005) 44(1):32–9. 10.1093/rheumatology/keh406 15367748

[55] Codina R Vanasse A Kelekar A Vezys V Jemmerson R. Cytochrome c-induced lymphocyte death from the outside in: inhibition by serum leucine-rich alpha-2-glycoprotein-1. Apoptosis (2010) 15(2):139–52. 10.1007/s10495-009-0412-0 19851871

[56] Yoon KW Byun S Kwon E Hwang SY Chu K Hiraki M Control of signaling-mediated clearance of apoptotic cells by the tumor suppressor p53. Science (2015) 349(6247):1261669. 10.1126/science.1261669 26228159 PMC5215039

[57] Kao J Houck K Fan Y Haehnel I Libutti SK Kayton ML Characterization of a novel tumor-derived cytokine. Endothelial-monocyte activating polypeptide II. J Biol Chem (1994) 269(40):25106–19. 7929199

[58] Knies UE Behrensdorf HA Mitchell CA Deutsch U Risau W Drexler HC Regulation of endothelial monocyte-activating polypeptide II release by apoptosis. Proc Natl Acad Sci U S A (1998) 95(21):12322–7. 10.1073/pnas.95.21.12322 9770485 PMC22830

[59] Ahrens S Zelenay S Sancho D Hanc P Kjaer S Feest C F-actin is an evolutionarily conserved damage-associated molecular pattern recognized by DNGR-1, a receptor for dead cells. Immunity (2012) 36(4):635–45. 10.1016/j.immuni.2012.03.008 22483800

[60] Chiba S Baghdadi M Akiba H Yoshiyama H Kinoshita I Dosaka-Akita H Tumor-infiltrating DCs suppress nucleic acid-mediated innate immune responses through interactions between the receptor TIM-3 and the alarmin HMGB1. Nat Immunol (2012) 13(9):832–42. 10.1038/ni.2376 22842346 PMC3622453

[61] Zitvogel L Kepp O Kroemer G. Decoding cell death signals in inflammation and immunity. Cell (2010) 140(6):798–804. 10.1016/j.cell.2010.02.015 20303871

[62] Pletjushkina OY Fetisova EK Lyamzaev KG Ivanova OY Domnina LV Vyssokikh MY Long-distance apoptotic killing of cells is mediated by hydrogen peroxide in a mitochondrial ROS-dependent fashion. Cell Death Differ (2005) 12(11):1442–4. 10.1038/sj.cdd.4401685 15933738

[63] Garg AD Nowis D Golab J Agostinis P. Photodynamic therapy: illuminating the road from cell death towards anti-tumour immunity. Apoptosis (2010) 15(9):1050–71. 10.1007/s10495-010-0479-7 20221698

[64] Suzuki S Kulkarni AB. Extracellular heat shock protein HSP90beta secreted by MG63 osteosarcoma cells inhibits activation of latent TGF-beta1. Biochem Biophys Res Commun (2010) 398(3):525–31. 10.1016/j.bbrc.2010.06.112 20599762 PMC2922109

[65] Korbelik M Sun J Cecic I. Photodynamic therapy-induced cell surface expression and release of heat shock proteins: relevance for tumor response. Cancer Res (2005) 65(3):1018–26. 15705903

[66] Cirone M Di Renzo L Lotti LV Conte V Trivedi P Santarelli R Primary effusion lymphoma cell death induced by bortezomib and AG 490 activates dendritic cells through CD91. PLoS One (2012) 7(3):e31732. 10.1371/journal.pone.0031732 22412839 PMC3296697

[67] Zunino B Rubio-Patino C Villa E Meynet O Proics E Cornille A Hyperthermic intraperitoneal chemotherapy leads to an anticancer immune response via exposure of cell surface heat shock protein 90. Oncogene (2015). 10.1038/onc.2015.82 25867070

[68] Zhou Z Yamamoto Y Sugai F Yoshida K Kishima Y Sumi H Hepatoma-derived growth factor is a neurotrophic factor harbored in the nucleus. J Biol Chem (2004) 279(26):27320–6. 10.1074/jbc.M308650200 15140875

[69] Huang H Evankovich J Yan W Nace G Zhang L Ross M Endogenous histones function as alarmins in sterile inflammatory liver injury through toll-like receptor 9 in mice. Hepatology (2011) 54(3):999–1008. 10.1002/hep.24501 21721026 PMC3213322

[70] Apetoh L Ghiringhelli F Tesniere A Obeid M Ortiz C Criollo A Toll-like receptor 4-dependent contribution of the immune system to anticancer chemotherapy and radiotherapy. Nat Med (2007) 13(9):1050–9. 10.1038/nm1622 17704786

[71] Semino C Angelini G Poggi A Rubartelli A. NK/iDC interaction results in IL-18 secretion by DCs at the synaptic cleft followed by NK cell activation and release of the DC maturation factor HMGB1. Blood (2005) 106(2):609–16. 10.1182/blood-2004-10-3906 15802534

[72] Scaffidi P Misteli T Bianchi ME. Release of chromatin protein HMGB1 by necrotic cells triggers inflammation. Nature (2002) 418(6894):191–5. 10.1038/nature00858 12110890

[73] Thorburn J Horita H Redzic J Hansen K Frankel AE Thorburn A. Autophagy regulates selective HMGB1 release in tumor cells that are destined to die. Cell Death Differ (2009) 16(1):175–83. 10.1038/cdd.2008.143 18846108 PMC2605182

[74] Yang D Postnikov YV Li Y Tewary P de la Rosa G Wei F. High-mobility group nucleosome-binding protein 1 acts as an alarmin and is critical for lipopolysaccharide-induced immune responses. J Exp Med (2012) 209(1):157–71. 10.1084/jem.20101354 22184635 PMC3260868

[75] Cohen I Rider P Carmi Y Braiman A Dotan S White MR Differential release of chromatin-bound IL-1alpha discriminates between necrotic and apoptotic cell death by the ability to induce sterile inflammation. Proc Natl Acad Sci U S A (2010) 107(6):2574–9. 10.1073/pnas.0915018107 20133797 PMC2823886

[76] Vanden Berghe T Kalai M Denecker G Meeus A Saelens X Vandenabeele P. Necrosis is associated with IL-6 production but apoptosis is not. Cell Signal (2006) 18(3):328–35. 10.1016/j.cellsig.2005.05.003 16023831

[77] Lauber K Bohn E Krober SM Xiao YJ Blumenthal SG Lindemann RK Apoptotic cells induce migration of phagocytes via caspase-3-mediated release of a lipid attraction signal. Cell (2003) 113:717–30. 10.1016/S0092-8674(03)00422-7 12809603

[78] Zhang Q Raoof M Chen Y Sumi Y Sursal T Junger W Circulating mitochondrial DAMPs cause inflammatory responses to injury. Nature (2010) 464(7285):104–7. 10.1038/nature08780 20203610 PMC2843437

[79] Collins LV Hajizadeh S Holme E Jonsson IM Tarkowski A. Endogenously oxidized mitochondrial DNA induces in vivo and in vitro inflammatory responses. J Leukoc Biol (2004) 75(6):995–1000. 10.1189/jlb.0703328 14982943

[80] Galluzzi L Kepp O Kroemer G. Mitochondria: master regulators of danger signalling. Nat Rev Mol Cell Biol (2012) 13(12):780–8. 10.1038/nrm3479 23175281

[81] Shi Y Evans JE Rock KL. Molecular identification of a danger signal that alerts the immune system to dying cells. Nature (2003) 425(6957):516–21. 10.1038/nature01991 14520412

[82] Carp H. Mitochondrial N-formylmethionyl proteins as chemoattractants for neutrophils. J Exp Med (1982) 155(1):264–75. 10.1084/jem.155.1.264 6274994 PMC2186576

[83] Rabiet MJ Huet E Boulay F. The N-formyl peptide receptors and the anaphylatoxin C5a receptors: an overview. Biochimie (2007) 89(9):1089–106. 10.1016/j.biochi.2007.02.015 17428601 PMC7115771

[84] Czapiga M Gao JL Kirk A Lekstrom-Himes J. Human platelets exhibit chemotaxis using functional N-formyl peptide receptors. Exp Hematol (2005) 33(1):73–84. 10.1016/j.exphem.2004.09.010 15661400

[85] Moghaddam AE Gartlan KH Kong L Sattentau QJ. Reactive carbonyls are a major Th2-inducing damage-associated molecular pattern generated by oxidative stress. J Immunol (2011) 187(4):1626–33. 10.4049/jimmunol.1003906 21742965

[86] Miller YI Choi SH Wiesner P Fang L Harkewicz R Hartvigsen K Oxidation-specific epitopes are danger-associated molecular patterns recognized by pattern recognition receptors of innate immunity. Circ Res (2011) 108(2):235–48. 10.1161/CIRCRESAHA.110.223875 21252151 PMC3075542

[87] Vandenberk L Garg AD Verschuere T Koks C Belmans J Beullens M Irradiation of necrotic cancer cells employed for pulsing dendritic cells (DCs), potentiates DC vaccine-induced antitumor immunity against high-grade glioma. Oncoimmunology (2015). 10.1080/2162402X.2015.1083669 PMC4801426 27057467

[88] Riddell JR Wang XY Minderman H Gollnick SO. Peroxiredoxin 1 stimulates secretion of proinflammatory cytokines by binding to TLR4. J Immunol (2010) 184(2):1022–30. 10.4049/jimmunol.0901945 20018613 PMC2955897

[89] Franz S Herrmann K Furnrohr BG Sheriff A Frey B Gaipl US After shrinkage apoptotic cells expose internal membrane-derived epitopes on their plasma membranes. Cell Death Differ (2007) 14(4):733–42. 10.1038/sj.cdd.4402066 17170754

[90] Petrovski G Zahuczky G Katona K Vereb G Martinet W Nemes Z Clearance of dying autophagic cells of different origin by professional and non-professional phagocytes. Cell Death Differ (2007) 14(6):1117–28. 10.1038/sj.cdd.4402112 17363964

[91] Bratton DL Fadok VA Richter DA Kailey JM Guthrie LA Henson PM. Appearance of phosphatidylserine on apoptotic cells requires calcium-mediated nonspecific flip-flop and is enhanced by loss of the aminophospholipid translocase. J Biol Chem (1997) 272(42):26159–65. 10.1074/jbc.272.42.26159 9334182

[92] Martin SJ Reutelingsperger CP McGahon AJ Rader JA van Schie RC LaFace DM Early redistribution of plasma membrane phosphatidylserine is a general feature of apoptosis regardless of the initiating stimulus: inhibition by overexpression of Bcl-2 and Abl. J Exp Med (1995) 182(5):1545–56. 10.1084/jem.182.5.1545 7595224 PMC2192182

[93] Brouckaert G Kalai M Krysko DV Saelens X Vercammen D Ndlovu MN Phagocytosis of necrotic cells by macrophages is phosphatidylserine dependent and does not induce inflammatory cytokine production. Mol Biol Cell (2004) 15(3):1089–100. 10.1091/mbc.E03-09-0668 14668480 PMC363082

[94] Donato R. RAGE: a single receptor for several ligands and different cellular responses: the case of certain S100 proteins. Curr Mol Med (2007) 7(8):711–24. 10.2174/156652407783220688 18331229

[95] Goh FG Piccinini AM Krausgruber T Udalova IA Midwood KS. Transcriptional regulation of the endogenous danger signal tenascin-C: a novel autocrine loop in inflammation. J Immunol (2010) 184(5):2655–62. 10.4049/jimmunol.0903359 20107185

[96] Krispin A Bledi Y Atallah M Trahtemberg U Verbovetski I Nahari E Apoptotic cell thrombospondin-1 and heparin-binding domain lead to dendritic-cell phagocytic and tolerizing states. Blood (2006) 108(10):3580–9. 10.1182/blood-2006-03-013334 16882710

[97] Galluzzi L Bravo-San Pedro JM Vitale I Aaronson SA Abrams JM Adam D Essential versus accessory aspects of cell death: recommendations of the NCCD 2015. Cell Death Differ (2015) 22(1):58–73. 10.1038/cdd.2014.137 25236395 PMC4262782

[98] Melcher A Todryk S Hardwick N Ford M Jacobson M Vile RG. Tumor immunogenicity is determined by the mechanism of cell death via induction of heat shock protein expression. Nat Med (1998) 4(5):581–7. 10.1038/nm0598-581 9585232

[99] Gough MJ Melcher AA Crittenden MR Sanchez-Perez L Voellmy R Vile RG. Induction of cell stress through gene transfer of an engineered heat shock transcription factor enhances tumor immunogenicity. Gene Ther (2004) 11(13):1099–104. 10.1038/sj.gt.3302274 15103319

[100] Spisek R Charalambous A Mazumder A Vesole DH Jagannath S Dhodapkar MV. Bortezomib enhances dendritic cell (DC)-mediated induction of immunity to human myeloma via exposure of cell surface heat shock protein 90 on dying tumor cells: therapeutic implications. Blood (2007) 109(11):4839–45. 10.1182/blood-2006-10-054221 17299090 PMC1885516

[101] Casares N Pequignot MO Tesniere A Ghiringhelli F Roux S Chaput N Caspase-dependent immunogenicity of doxorubicin-induced tumor cell death. J Exp Med (2005) 202(12):1691–701. 10.1084/jem.20050915 16365148 PMC2212968

[102] Kroemer G Galluzzi L Kepp O Zitvogel L Immunogenic cell death in cancer therapy. Annu Rev Immunol (2013) 31:51–72. 10.1146/annurev-immunol-032712-100008 23157435

[103] Garg AD Dudek-Peric AM Romano E Agostinis P. Immunogenic cell death. Int J Dev Biol (2015) 59:131–40. 10.1387/ijdb.150061pa 26374534

[104] Kepp O Senovilla L Vitale I Vacchelli E Adjemian S Agostinis P Consensus guidelines for the detection of immunogenic cell death. Oncoimmunology (2014) 3(9):e955691. 10.4161/21624011.2014.955691 25941621 PMC4292729

[105] Dudek AM Martin S Garg AD Agostinis P. Immature, semi-mature, and fully mature dendritic cells: toward a DC-cancer cells interface that augments anticancer immunity. Front Immunol (2014) 4:438. 10.3389/fimmu.2013.00438 24376443 PMC3858649

[106] Ma Y Adjemian S Mattarollo SR Yamazaki T Aymeric L Yang H Anticancer chemotherapy-induced intratumoral recruitment and differentiation of antigen-presenting cells. Immunity (2013) 38(4):729–41. 10.1016/j.immuni.2013.03.003 23562161

[107] Zhang JG Czabotar PE Policheni AN Caminschi I Wan SS Kitsoulis S The dendritic cell receptor Clec9A binds damaged cells via exposed actin filaments. Immunity (2012) 36(4):646–57. 10.1016/j.immuni.2012.03.009 22483802

[108] Garnett CT Palena C Chakraborty M Tsang KY Schlom J Hodge JW. Sublethal irradiation of human tumor cells modulates phenotype resulting in enhanced killing by cytotoxic T lymphocytes. Cancer Res (2004) 64(21):7985–94. 10.1158/0008-5472.CAN-04-1525 15520206

[109] Hodge JW Garnett CT Farsaci B Palena C Tsang KY Ferrone S Chemotherapy-induced immunogenic modulation of tumor cells enhances killing by cytotoxic T lymphocytes and is distinct from immunogenic cell death. Int J Cancer (2013) 133(3):624–36. 10.1002/ijc.28070 23364915 PMC3663913

[110] Gameiro SR Jammeh ML Wattenberg MM Tsang KY Ferrone S Hodge JW. Radiation-induced immunogenic modulation of tumor enhances antigen processing and calreticulin exposure, resulting in enhanced T-cell killing. Oncotarget (2014) 5(2):403–16. 10.18632/oncotarget.1719 24480782 PMC3964216

[111] Michaud M Martins I Sukkurwala AQ Adjemian S Ma Y Pellegatti P Autophagy-dependent anticancer immune responses induced by chemotherapeutic agents in mice. Science (2011) 334(6062):1573–7. 10.1126/science.1208347 22174255

[112] Garg AD Dudek AM Agostinis P Calreticulin surface exposure is abrogated in cells lacking, chaperone-mediated autophagy-essential gene, LAMP2A. Cell Death Dis (2013) 4:e826 10.1038/cddis.2013.372 24091669 PMC3824681

[113] Martins I Wang Y Michaud M Ma Y Sukkurwala AQ Shen S Molecular mechanisms of ATP secretion during immunogenic cell death. Cell Death Differ (2014) 21(1):79–91. 10.1038/cdd.2013.75 23852373 PMC3857631

[114] Kazama H Ricci JE Herndon JM Hoppe G Green DR Ferguson TA. Induction of immunological tolerance by apoptotic cells requires caspase-dependent oxidation of high-mobility group box-1 protein. Immunity (2008) 29(1):21–32. 10.1016/j.immuni.2008.05.013 18631454 PMC2704496

[115] Jube S Rivera Z Bianchi ME Powers A Wang E Pagano IS Cancer cell secretion of the DAMP protein HMGB1 supports progression in malignant mesothelioma. Cancer Res (2012) 72(13):3290–301. 10.1158/0008-5472.CAN-11-3481 22552293 PMC3389268

[116] Garg AD Krysko DV Vandenabeele P Agostinis P. Hypericin-based photodynamic therapy induces surface exposure of damage-associated molecular patterns like HSP70 and calreticulin. Cancer Immunol Immunother (2012) 61(2):215–21. 10.1007/s00262-011-1184-2 22193987 PMC11029694

[117] Lancaster GI Febbraio MA. Exosome-dependent trafficking of HSP70: a novel secretory pathway for cellular stress proteins. J Biol Chem (2005) 280(24):23349–55. 10.1074/jbc.M502017200 15826944

[118] Mambula SS Calderwood SK. Heat shock protein 70 is secreted from tumor cells by a nonclassical pathway involving lysosomal endosomes. J Immunol (2006) 177(11):7849–57. 10.4049/jimmunol.177.11.7849 17114456

[119] Vega VL Rodriguez-Silva M Frey T Gehrmann M Diaz JC Steinem C Hsp70 translocates into the plasma membrane after stress and is released into the extracellular environment in a membrane-associated form that activates macrophages. J Immunol (2008) 180(6):4299–307. 10.4049/jimmunol.180.6.4299 18322243

[120] Kotter B Frey B Winderl M Rubner Y Scheithauer H Sieber R The in vitro immunogenic potential of caspase-3 proficient breast cancer cells with basal low immunogenicity is increased by hypofractionated irradiation. Radiat Oncol (2015) 10(1):197. 10.1186/s13014-015-0506-5 26383236 PMC4573696

[121] Multhoff G Botzler C Wiesnet M Muller E Meier T Wilmanns W A stress-inducible 72-kDa heat-shock protein (HSP72) is expressed on the surface of human tumor cells, but not on normal cells. Int J Cancer (1995) 61(2):272–9. 10.1002/ijc.2910610222 7705958

[122] Gastpar R Gehrmann M Bausero MA Asea A Gross C Schroeder JA Heat shock protein 70 surface-positive tumor exosomes stimulate migratory and cytolytic activity of natural killer cells. Cancer Res (2005) 65(12):5238–47. 10.1158/0008-5472.CAN-04-3804 15958569 PMC1785299

[123] Panaretakis T Kepp O Brockmeier U Tesniere A Bjorklund AC Chapman DC Mechanisms of pre-apoptotic calreticulin exposure in immunogenic cell death. EMBO J (2009) 28(5):578–90. 10.1038/emboj.2009.1 19165151 PMC2657583

[124] Madeo F Durchschlag M Kepp O Panaretakis T Zitvogel L Frohlich KU Phylogenetic conservation of the preapoptotic calreticulin exposure pathway from yeast to mammals. Cell Cycle (2009) 8(4):639–42. 10.4161/cc.8.4.7794 19182525

[125] Martin S Dudek-Peric AM Maes H Garg AD Gabrysiak M Demirsoy S Concurrent MEK and autophagy inhibition is required to restore cell death associated danger-signalling in vemurafenib-resistant melanoma cells. Biochem Pharmacol (2015) 93(3):290–304. 10.1016/j.bcp.2014.12.003 25529535

[126] Szklarczyk D Franceschini A Wyder S Forslund K Heller D Huerta-Cepas J STRING v10: protein-protein interaction networks, integrated over the tree of life. Nucleic Acids Res (2015) 43(Database issue):D447–52. 10.1093/nar/gku1003 25352553 PMC4383874

[127] Dudek AM Garg AD Krysko DV De Ruysscher D Agostinis P. Inducers of immunogenic cancer cell death. Cytokine Growth Factor Rev (2013) 24(4):319–33. 10.1016/j.cytogfr.2013.01.005 23391812

[128] Bezu L Gomes-de-Silva LC Dewitte H Breckpot K Fucikova J Spisek R Combinatorial strategies for the induction of immunogenic cell death. Front Immunol (2015) 6:187. 10.3389/fimmu.2015.00187 25964783 PMC4408862

[129] Siurala M Bramante S Vassilev L Hirvinen M Parviainen S Tahtinen S Oncolytic adenovirus and doxorubicin-based chemotherapy results in synergistic antitumor activity against soft-tissue sarcoma. Int J Cancer (2015) 136(4):945–54. 10.1002/ijc.29048 24975392

[130] Menger L Vacchelli E Adjemian S Martins I Ma Y Shen S Cardiac glycosides exert anticancer effects by inducing immunogenic cell death. Sci Transl Med (2012) 4(143):143ra99. 10.1126/scitranslmed.3003807 22814852

[131] Panaretakis T Joza N Modjtahedi N Tesniere A Vitale I Durchschlag M The co-translocation of ERp57 and calreticulin determines the immunogenicity of cell death. Cell Death Differ (2008) 15(9):1499–509. 10.1038/cdd.2008.67 18464797

[132] Tufi R Panaretakis T Bianchi K Criollo A Fazi B Di Sano F Reduction of endoplasmic reticulum Ca2+ levels favors plasma membrane surface exposure of calreticulin. Cell Death Differ (2008) 15(2):274–82. 10.1038/sj.cdd.4402275 18034188

[133] Martins I Kepp O Schlemmer F Adjemian S Tailler M Shen S Restoration of the immunogenicity of cisplatin-induced cancer cell death by endoplasmic reticulum stress. Oncogene (2011) 30(10):1147–58. 10.1038/onc.2010.500 21151176

[134] Verfaillie T Rubio N Garg AD Bultynck G Rizzuto R Decuypere JP PERK is required at the ER-mitochondrial contact sites to convey apoptosis after ROS-based ER stress. Cell Death Differ (2012) 19(11):1880–91. 10.1038/cdd.2012.74 22705852 PMC3469056

[135] Galluzzi L Bravo-San Pedro JM Kroemer G. Organelle-specific initiation of cell death. Nat Cell Biol (2014) 16(8):728–36. 10.1038/ncb3005 25082195

[136] Chaurio RA Munoz LE Maueroder C Janko C Harrer T Furnrohr BG The progression of cell death affects the rejection of allogeneic tumors in immune-competent mice – implications for cancer therapy. Front Immunol (2014) 5:560. 10.3389/fimmu.2014.00560 25426116 PMC4227513

[137] Garg AD Maes H van Vliet AR Agostinis P Targeting the hallmarks of cancer with therapy-induced endoplasmic reticulum (ER) stress. Mol Cell Oncol (2015) 2(1):e975089 10.4161/23723556.2014.975089 PMC4905250 27308392

[138] van Vliet AR Martin S Garg AD Agostinis P. The PERKs of damage-associated molecular patterns mediating cancer immunogenicity: from sensor to the plasma membrane and beyond. Semin Cancer Biol (2015) 33:74–85. 10.1016/j.semcancer.2015.03.010 25882379

[139] Sukkurwala AQ Adjemian S Senovilla L Michaud M Spaggiari S Vacchelli E Screening of novel immunogenic cell death inducers within the NCI mechanistic diversity set. Oncoimmunology (2014) 3:e28473. 10.4161/onci.28473 25050214 PMC4063139

[140] Wong DY Ong WW Ang WH. Induction of immunogenic cell death by chemotherapeutic platinum complexes. Angew Chem Int Ed Engl (2015) 54(22):6483–7. 10.1002/anie.201500934 25873535

[141] Sistigu A Yamazaki T Vacchelli E Chaba K Enot DP Adam J Cancer cell-autonomous contribution of type I interferon signaling to the efficacy of chemotherapy. Nat Med (2014) 20(11):1301–9. 10.1038/nm.3708 25344738

[142] Ramakrishnan R Gabrilovich DI. The role of mannose-6-phosphate receptor and autophagy in influencing the outcome of combination therapy. Autophagy (2013) 9(4):615–6. 10.4161/auto.23485 23324210 PMC3627678

[143] Kaminski JM Shinohara E Summers JB Niermann KJ Morimoto A Brousal J. The controversial abscopal effect. Cancer Treat Rev (2005) 31(3):159–72. 10.1016/j.ctrv.2005.03.004 15923088

[144] Golden EB Frances D Pellicciotta I Demaria S Helen Barcellos-Hoff M Formenti SC. Radiation fosters dose-dependent and chemotherapy-induced immunogenic cell death. Oncoimmunology (2014) 3:e28518. 10.4161/onci.28518 25071979 PMC4106151

[145] Garrido G Rabasa A Sanchez B Lopez MV Blanco R Lopez A Induction of immunogenic apoptosis by blockade of epidermal growth factor receptor activation with a specific antibody. J Immunol (2011) 187(10):4954–66. 10.4049/jimmunol.1003477 21984704

[146] Bugaut H Bruchard M Berger H Derangere V Odoul L Euvrard R Bleomycin exerts ambivalent antitumor immune effect by triggering both immunogenic cell death and proliferation of regulatory T cells. PLoS One (2013) 8(6):e65181. 10.1371/journal.pone.0065181 23762310 PMC3676388

[147] Diaconu I Cerullo V Hirvinen ML Escutenaire S Ugolini M Pesonen SK Immune response is an important aspect of the antitumor effect produced by a CD40L-encoding oncolytic adenovirus. Cancer Res (2012) 72(9):2327–38. 10.1158/0008-5472.CAN-11-2975 22396493

[148] Hemminki O Parviainen S Juhila J Turkki R Linder N Lundin J Immunological data from cancer patients treated with Ad5/3-E2F-Delta24-GMCSF suggests utility for tumor immunotherapy. Oncotarget (2015) 6(6):4467–81. 10.18632/oncotarget.2901 25714011 PMC4414204

[149] Sun C Wang H Mao S Liu J Li S Wang J. Reactive oxygen species involved in CT26 immunogenic cell death induced by *Clostridium difficile* toxin B. Immunol Lett (2015) 164(2):65–71. 10.1016/j.imlet.2015.02.007 25721381

[150] Miyamoto S Inoue H Nakamura T Yamada M Sakamoto C Urata Y *Coxsackievirus* B3 Is an oncolytic virus with immunostimulatory properties that is active against lung Adenocarcinoma. Cancer Res (2012) 72(10):2609–21. 10.1158/0008-5472.CAN-11-3185 22461509

[151] Vacchelli E Eggermont A Sautes-Fridman C Galon J Zitvogel L Kroemer G Trial watch: oncolytic viruses for cancer therapy. Oncoimmunology (2013) 2(6):e24612. 10.4161/onci.22789 23894720 PMC3716755

[152] Schiavoni G Sistigu A Valentini M Mattei F Sestili P Spadaro F Cyclophosphamide synergizes with type I interferons through systemic dendritic cell reactivation and induction of immunogenic tumor apoptosis. Cancer Res (2011) 71(3):768–78. 10.1158/0008-5472.CAN-10-2788 21156650

[153] Viaud S Saccheri F Mignot G Yamazaki T Daillere R Hannani D The intestinal microbiota modulates the anticancer immune effects of cyclophosphamide. Science (2013) 342(6161):971–6. 10.1126/science.1240537 24264990 PMC4048947

[154] Fucikova J Moserova I Truxova I Hermanova I Vancurova I Partlova S High hydrostatic pressure induces immunogenic cell death in human tumor cells. Int J Cancer (2014) 135(5):1165–77. 10.1002/ijc.28766 24500981

[155] Adkins I Fucikova J Garg AD Agostinis P Spisek R. Physical modalities inducing immunogenic tumor cell death for cancer immunotherapy. Oncoimmunology (2014) 3:e968434. 10.4161/21624011.2014.968434 25964865 PMC4352954

[156] Weiss EM Meister S Janko C Ebel N Schlucker E Meyer-Pittroff R High hydrostatic pressure treatment generates inactivated mammalian tumor cells with immunogeneic features. J Immunotoxicol (2010) 7(3):194–204. 10.3109/15476911003657414 20205624

[157] Garg AD Agostinis P. ER stress, autophagy and immunogenic cell death in photodynamic therapy-induced anti-cancer immune responses. Photochem Photobiol Sci (2014) 13(3):474–87. 10.1039/c3pp50333j 24493131

[158] Yu Z Geng J Zhang M Zhou Y Fan Q Chen J. Treatment of osteosarcoma with microwave thermal ablation to induce immunogenic cell death. Oncotarget (2014) 5(15):6526–39. 10.18632/oncotarget.2310 25153727 PMC4171648

[159] Zamarin D Holmgaard RB Subudhi SK Park JS Mansour M Palese P Localized oncolytic virotherapy overcomes systemic tumor resistance to immune checkpoint blockade immunotherapy. Sci Transl Med (2014) 6(226):226ra32. 10.1126/scitranslmed.3008095 24598590 PMC4106918

[160] Chen HM Wang PH Chen SS Wen CC Chen YH Yang WC Shikonin induces immunogenic cell death in tumor cells and enhances dendritic cell-based cancer vaccine. Cancer Immunol Immunother (2012) 61(11):1989–2002. 10.1007/s00262-012-1258-9 22527248 PMC11029192

[161] Korbelik M Dougherty GJ. Photodynamic therapy-mediated immune response against subcutaneous mouse tumors. Cancer Res (1999) 59(8):1941–6. 10213504

[162] Krosl G Korbelik M Dougherty GJ. Induction of immune cell infiltration into murine SCCVII tumour by photofrin-based photodynamic therapy. Br J Cancer (1995) 71(3):549–55. 10.1038/bjc.1995.108 7880738 PMC2033617

[163] Korbelik M Stott B Sun J. Photodynamic therapy-generated vaccines: relevance of tumour cell death expression. Br J Cancer (2007) 97(10):1381–7. 10.1038/sj.bjc.6604059 17971767 PMC2360230

[164] Korbelik M Zhang W Merchant S. Involvement of damage-associated molecular patterns in tumor response to photodynamic therapy: surface expression of calreticulin and high-mobility group box-1 release. Cancer Immunol Immunother (2011) 60(10):1431–7. 10.1007/s00262-011-1047-x 21644033 PMC11028986

[165] Duewell P Steger A Lohr H Bourhis H Hoelz H Kirchleitner SV RIG-I-like helicases induce immunogenic cell death of pancreatic cancer cells and sensitize tumors toward killing by CD8 T cells. Cell Death Differ (2014) 21(12):1825–37. 10.1038/cdd.2014.96 25012502 PMC4227156

[166] West AC Mattarollo SR Shortt J Cluse LA Christiansen AJ Smyth MJ An intact immune system is required for the anticancer activities of histone deacetylase inhibitors. Cancer Res (2013) 73(24):7265–76. 10.1158/0008-5472.CAN-13-0890 24158093

[167] Yang Y Li XJ Chen Z Zhu XX Wang J Zhang LB Wogonin induced calreticulin/annexin A1 exposure dictates the immunogenicity of cancer cells in a PERK/AKT dependent manner. PLoS One (2012) 7(12):e50811. 10.1371/journal.pone.0050811 23251389 PMC3520942

[168] Panzarini E Inguscio V Fimia GM Dini L. Rose Bengal acetate photodynamic therapy (RBAc-PDT) induces exposure and release of damage-associated molecular patterns (DAMPs) in human HeLa cells. PLoS One (2014) 9(8):e105778. 10.1371/journal.pone.0105778 25140900 PMC4139382

[169] Molinari R D’Eliseo D Manzi L Zolla L Velotti F Merendino N. The n3-polyunsaturated fatty acid docosahexaenoic acid induces immunogenic cell death in human cancer cell lines via pre-apoptotic calreticulin exposure. Cancer Immunol Immunother (2011) 60(10):1503–7. 10.1007/s00262-011-1074-7 21779875 PMC11028828

[170] D’Eliseo D Manzi L Velotti F. Capsaicin as an inducer of damage-associated molecular patterns (DAMPs) of immunogenic cell death (ICD) in human bladder cancer cells. Cell Stress Chaperones (2013) 18(6):801–8. 10.1007/s12192-013-0422-2 23580156 PMC3789874

[171] Gilardini Montani MS D’Eliseo D Cirone M Di Renzo L Faggioni A Santoni A Capsaicin-mediated apoptosis of human bladder cancer cells activates dendritic cells via CD91. Nutrition (2015) 31(4):578–81. 10.1016/j.nut.2014.05.005 25220876

[172] Janeway C Immunobiology: The Immune System in Health and Disease. 6th ed New York, NY: Garland Science (2005). 823 p.

[173] Fucikova J Kralikova P Fialova A Brtnicky T Rob L Bartunkova J Human tumor cells killed by anthracyclines induce a tumor-specific immune response. Cancer Res (2011) 71(14):4821–33. 10.1158/0008-5472.CAN-11-0950 21602432

[174] Krysko DV Kaczmarek A Krysko O Heyndrickx L Woznicki J Bogaert P TLR-2 and TLR-9 are sensors of apoptosis in a mouse model of doxorubicin-induced acute inflammation. Cell Death Differ (2011) 18(8):1316–25. 10.1038/cdd.2011.4 21311566 PMC3172099

[175] Tseng LM Liu CY Chang KC Chu PY Shiau CW Chen KF. CIP2A is a target of bortezomib in human triple negative breast cancer cells. Breast Cancer Res (2012) 14(2):R68. 10.1186/bcr3175 22537901 PMC3446403

[176] Davies AM Lara PN Jr Mack PC Gandara DR. Incorporating bortezomib into the treatment of lung cancer. Clin Cancer Res (2007) 13(15 Pt 2):s4647–51. 10.1158/1078-0432.CCR-07-0334 17671158

[177] Huang T Li S Li G Tian Y Wang H Shi L Utility of *Clostridium difficile* toxin B for inducing anti-tumor immunity. PLoS One (2014) 9(10):e110826. 10.1371/journal.pone.0110826 25340750 PMC4207755

[178] Bravim F de Freitas JM Fernandes AA Fernandes PM. High hydrostatic pressure and the cell membrane: stress response of *Saccharomyces cerevisiae*. Ann N Y Acad Sci (2010) 1189:127–32. 10.1111/j.1749-6632.2009.05182.x 20233378

[179] Senovilla L Vitale I Martins I Tailler M Pailleret C Michaud M An immunosurveillance mechanism controls cancer cell ploidy. Science (2012) 337(6102):1678–84. 10.1126/science.1224922 23019653

[180] Korbelik M. Cancer vaccines generated by photodynamic therapy. Photochem Photobiol Sci (2011) 10(5):664–9. 10.1039/c0pp00343c 21258728

[181] Garg AD Krysko DV Vandenabeele P Agostinis P. DAMPs and PDT-mediated photo-oxidative stress: exploring the unknown. Photochem Photobiol Sci (2011) 10(5):670–80. 10.1039/c0pp00294a 21258717

[182] Chen J Xie J Jiang Z Wang B Wang Y Hu X. Shikonin and its analogs inhibit cancer cell glycolysis by targeting tumor pyruvate kinase-M2. Oncogene (2011) 30(42):4297–306. 10.1038/onc.2011.137 21516121

[183] Tsai CF Yeh WL Huang SM Tan TW Lu DY. Wogonin induces reactive oxygen species production and cell apoptosis in human glioma cancer cells. Int J Mol Sci (2012) 13(8):9877–92. 10.3390/ijms13089877 22949836 PMC3431834

[184] Sanovic R Verwanger T Hartl A Krammer B. Low dose hypericin-PDT induces complete tumor regression in BALB/c mice bearing CT26 colon carcinoma. Photodiagnosis Photodyn Ther (2011) 8(4):291–6. 10.1016/j.pdpdt.2011.04.003 22122915

[185] Garg AD Krysko DV Vandenabeele P Agostinis P. The emergence of phox-ER stress induced immunogenic apoptosis. OncoImmunology (2012) 1(5):787–9. 10.4161/onci.19750 22934283 PMC3429595

[186] Liu Z Zhang HM Yuan J Ye X Taylor GA Yang D. The immunity-related GTPase Irgm3 relieves endoplasmic reticulum stress response during *Coxsackievirus* B3 infection via a PI3K/Akt dependent pathway. Cell Microbiol (2012) 14(1):133–46. 10.1111/j.1462-5822.2011.01708.x 21981022 PMC3691006

[187] Bian J Wang K Kong X Liu H Chen F Hu M Caspase- and p38-MAPK-dependent induction of apoptosis in A549 lung cancer cells by Newcastle disease virus. Arch Virol (2011) 156(8):1335–44. 10.1007/s00705-011-0987-y 21625975

[188] Garg AD De Ruysscher D Agostinis P Immunological metagene signatures derived from immunogenic cancer cell death associate with improved survival of patients with lung, breast or ovarian malignancies: a large-scale meta-analysis. Oncoimmunology (2015). 10.1080/2162402X.2015.1069938 PMC4801472 27057433

[189] Galluzzi L Kepp O Kroemer G Enlightening the impact of immunogenic cell death in photodynamic cancer therapy. EMBO J (2012) 31(5):1055–7. 10.1038/emboj.2012.2 22252132 PMC3298006

[190] Galluzzi L Pietrocola F Bravo-San Pedro JM Amaravadi RK Baehrecke EH Cecconi F Autophagy in malignant transformation and cancer progression. EMBO J (2015) 34(7):856–80. 10.15252/embj.201490784 25712477 PMC4388596

[191] Mattarollo SR Loi S Duret H Ma Y Zitvogel L Smyth MJ. Pivotal role of innate and adaptive immunity in anthracycline chemotherapy of established tumors. Cancer Res (2011) 71(14):4809–20. 10.1158/0008-5472.CAN-11-0753 21646474

[192] Lin TJ Lin HT Chang WT Mitapalli SP Hsiao PW Yin SY Shikonin-enhanced cell immunogenicity of tumor vaccine is mediated by the differential effects of DAMP components. Mol Cancer (2015) 14:174. 10.1186/s12943-015-0435-9 26403780 PMC4582891

[193] Ma Y Aymeric L Locher C Mattarollo SR Delahaye NF Pereira P Contribution of IL-17-producing gamma delta T cells to the efficacy of anticancer chemotherapy. J Exp Med (2011) 208(3):491–503. 10.1084/jem.20100269 21383056 PMC3058575

[194] Yang H Yamazaki T Pietrocola F Zhou H Zitvogel L Ma Y STAT3 inhibition enhances the therapeutic efficacy of immunogenic chemotherapy by stimulating type 1 interferon production by cancer cells. Cancer Res (2015) 75(18):3812–22. 10.1158/0008-5472.CAN-15-1122 26208907

[195] Ciampricotti M Hau CS Doornebal CW Jonkers J de Visser KE Chemotherapy response of spontaneous mammary tumors is independent of the adaptive immune system. Nat Med (2012) 18(3):344–6. 10.1038/nm.2652 22395693

[196] Gould SE Junttila MR de Sauvage FJ. Translational value of mouse models in oncology drug development. Nat Med (2015) 21(5):431–9. 10.1038/nm.3853 25951530

[197] Tesniere A Schlemmer F Boige V Kepp O Martins I Ghiringhelli F Immunogenic death of colon cancer cells treated with oxaliplatin. Oncogene (2010) 29(4):482–91. 10.1038/onc.2009.356 19881547

[198] Hannesdottir L Tymoszuk P Parajuli N Wasmer MH Philipp S Daschil N Lapatinib and doxorubicin enhance the Stat1-dependent antitumor immune response. Eur J Immunol (2013) 43(10):2718–29. 10.1002/eji.201242505 23843024

[199] Michaud M Xie X Bravo-San Pedro JM Zitvogel L White E Kroemer G. An autophagy-dependent anticancer immune response determines the efficacy of melanoma chemotherapy. Oncoimmunology (2014) 3(7):e944047. 10.4161/21624011.2014.944047 25610726 PMC4292732

[200] Demaria S Santori FR Ng B Liebes L Formenti SC Vukmanovic S. Select forms of tumor cell apoptosis induce dendritic cell maturation. J Leukoc Biol (2005) 77(3):361–8. 10.1189/jlb.0804478 15569694

[201] Schumacher LY Vo DD Garban HJ Comin-Anduix B Owens SK Dissette VB Immunosensitization of tumor cells to dendritic cell-activated immune responses with the proteasome inhibitor bortezomib (PS-341, Velcade). J Immunol (2006) 176(8):4757–65. 10.4049/jimmunol.176.8.4757 16585569

[202] Chang CL Hsu YT Wu CC Yang YC Wang C Wu TC Immune mechanism of the antitumor effects generated by bortezomib. J Immunol (2012) 189(6):3209–20. 10.4049/jimmunol.1103826 22896634

[203] van der Most RG Currie AJ Mahendran S Prosser A Darabi A Robinson BW Tumor eradication after cyclophosphamide depends on concurrent depletion of regulatory T cells: a role for cycling TNFR2-expressing effector-suppressor T cells in limiting effective chemotherapy. Cancer Immunol Immunother (2009) 58(8):1219–28. 10.1007/s00262-008-0628-9 19052741 PMC11030690

[204] Obeid M Panaretakis T Joza N Tufi R Tesniere A van Endert P Calreticulin exposure is required for the immunogenicity of gamma-irradiation and UVC light-induced apoptosis. Cell Death Differ (2007) 14(10):1848–50. 10.1038/sj.cdd.4402201 17657249

[205] Carr-Brendel V Markovic D Smith M Taylor-Papadimitriou J Cohen EP. Immunity to breast cancer in mice immunized with X-irradiated breast cancer cells modified to secrete IL-12. J Immunother (1999) 22(5):415–22. 10.1097/00002371-199909000-00005 10546157

[206] Strome SE Voss S Wilcox R Wakefield TL Tamada K Flies D Strategies for antigen loading of dendritic cells to enhance the antitumor immune response. Cancer Res (2002) 62(6):1884–9. 11912169

[207] Prasad SJ Farrand KJ Matthews SA Chang JH McHugh RS Ronchese F. Dendritic cells loaded with stressed tumor cells elicit long-lasting protective tumor immunity in mice depleted of CD4+CD25+ regulatory T cells. J Immunol (2005) 174(1):90–8. 10.4049/jimmunol.174.1.90 15611231

[208] Yang H Zhou P Huang H Chen D Ma N Cui QC Shikonin exerts antitumor activity via proteasome inhibition and cell death induction in vitro and in vivo. Int J Cancer (2009) 124(10):2450–9. 10.1002/ijc.24195 19165859 PMC2707765

[209] Sharma P Allison JP. The future of immune checkpoint therapy. Science (2015) 348(6230):56–61. 10.1126/science.aaa8172 25838373

[210] Viney M Lazarou L Abolins S. The laboratory mouse and wild immunology. Parasite Immunol (2015) 37(5):267–73. 10.1111/pim.12150 25303494

[211] Davis MM. A prescription for human immunology. Immunity (2008) 29(6):835–8. 10.1016/j.immuni.2008.12.003 19100694 PMC2905652

[212] Mestas J Hughes CC. Of mice and not men: differences between mouse and human immunology. J Immunol (2004) 172(5):2731–8. 10.4049/jimmunol.172.5.2731 14978070

[213] Tubiana M. Klaas Breur medal lecture 1985. The growth and progression of human tumors: implications for management strategy. Radiother Oncol (1986) 6(3):167–84. 10.1016/S0167-8140(86)80151-7 3529254

[214] Klein CA. Parallel progression of primary tumours and metastases. Nat Rev Cancer (2009) 9(4):302–12. 10.1038/nrc2627 19308069

[215] Yamazaki T Hannani D Poirier-Colame V Ladoire S Locher C Sistigu A Defective immunogenic cell death of HMGB1-deficient tumors: compensatory therapy with TLR4 agonists. Cell Death Differ (2014) 21(1):69–78. 10.1038/cdd.2013.72 23811849 PMC3857617

[216] Loi S Pommey S Haibe-Kains B Beavis PA Darcy PK Smyth MJ CD73 promotes anthracycline resistance and poor prognosis in triple negative breast cancer. Proc Natl Acad Sci U S A (2013) 110(27):11091–6. 10.1073/pnas.1222251110 23776241 PMC3704029

[217] Shalapour S Font-Burgada J Di Caro G Zhong Z Sanchez-Lopez E Dhar D Immunosuppressive plasma cells impede T-cell-dependent immunogenic chemotherapy. Nature (2015) 521(7550):94–8. 10.1038/nature14395 25924065 PMC4501632

[218] De Boo S Kopecka J Brusa D Gazzano E Matera L Ghigo D iNOS activity is necessary for the cytotoxic and immunogenic effects of doxorubicin in human colon cancer cells. Mol Cancer (2009) 8:108. 10.1186/1476-4598-8-108 19925669 PMC2785770

[219] Riganti C Castella B Kopecka J Campia I Coscia M Pescarmona G Zoledronic acid restores doxorubicin chemosensitivity and immunogenic cell death in multidrug-resistant human cancer cells. PLoS One (2013) 8(4):e60975. 10.1371/journal.pone.0060975 23593363 PMC3625183

[220] Stoll G Enot D Mlecnik B Galon J Zitvogel L Kroemer G. Immune-related gene signatures predict the outcome of neoadjuvant chemotherapy. Oncoimmunology (2014) 3(1):e27884. 10.4161/onci.27884 24790795 PMC4004621

[221] Pardoll DM. The blockade of immune checkpoints in cancer immunotherapy. Nat Rev Cancer (2012) 12(4):252–64. 10.1038/nrc3239 22437870 PMC4856023

[222] Galon J Angell HK Bedognetti D Marincola FM. The continuum of cancer immunosurveillance: prognostic, predictive, and mechanistic signatures. Immunity (2013) 39(1):11–26. 10.1016/j.immuni.2013.07.008 23890060

[223] Fridman WH Pages F Sautes-Fridman C Galon J. The immune contexture in human tumours: impact on clinical outcome. Nat Rev Cancer (2012) 12(4):298–306. 10.1038/nrc3245 22419253

[224] Zitvogel L Tesniere A Kroemer G. Cancer despite immunosurveillance: immunoselection and immunosubversion. Nat Rev Immunol (2006) 6(10):715–27. 10.1038/nri1936 16977338

[225] Ladoire S Penault-Llorca F Senovilla L Dalban C Enot D Locher C Combined evaluation of LC3B puncta and HMGB1 expression predicts residual risk of relapse after adjuvant chemotherapy in breast cancer. Autophagy (2015) 11(10):1878–90. 10.1080/15548627.2015.1082022 26506894 PMC4824597

[226] Kacerovska D Pizinger K Majer F Smid F. Photodynamic therapy of nonmelanoma skin cancer with topical *Hypericum perforatum* extract – a pilot study. Photochem Photobiol (2008) 84(3):779–85. 10.1111/j.1751-1097.2007.00260.x 18179625

[227] Rook AH Wood GS Duvic M Vonderheid EC Tobia A Cabana B. A phase II placebo-controlled study of photodynamic therapy with topical hypericin and visible light irradiation in the treatment of cutaneous T-cell lymphoma and psoriasis. J Am Acad Dermatol (2010) 63(6):984–90. 10.1016/j.jaad.2010.02.039 20889234

[228] Koren H Schenk GM Jindra RH Alth G Ebermann R Kubin A Hypericin in phototherapy. J Photochem Photobiol B (1996) 36(2):113–9. 10.1016/S1011-1344(96)07357-5 9002247

[229] Alecu M Ursaciuc C Halalau F Coman G Merlevede W Waelkens E Photodynamic treatment of basal cell carcinoma and squamous cell carcinoma with hypericin. Anticancer Res (1998) 18(6B):4651–4. 9891535

[230] Liikanen I Koski A Merisalo-Soikkeli M Hemminki O Oksanen M Kairemo K Serum HMGB1 is a predictive and prognostic biomarker for oncolytic immunotherapy. Oncoimmunology (2015) 4(3):e989771. 10.4161/2162402X.2014.989771 25949903 PMC4404794

[231] Agostinis P Berg K Cengel KA Foster TH Girotti AW Gollnick SO Photodynamic therapy of cancer: an update. CA Cancer J Clin (2011) 61(4):250–81. 10.3322/caac.20114 21617154 PMC3209659

[232] Suzuki Y Mimura K Yoshimoto Y Watanabe M Ohkubo Y Izawa S Immunogenic tumor cell death induced by chemoradiotherapy in patients with esophageal squamous cell carcinoma. Cancer Res (2012) 72(16):3967–76. 10.1158/0008-5472.CAN-12-0851 22700877

[233] Zappasodi R Pupa SM Ghedini GC Bongarzone I Magni M Cabras AD Improved clinical outcome in indolent B-cell lymphoma patients vaccinated with autologous tumor cells experiencing immunogenic death. Cancer Res (2010) 70(22):9062–72. 10.1158/0008-5472.CAN-10-1825 20884630

[234] Baitsch L Fuertes-Marraco SA Legat A Meyer C Speiser DE. The three main stumbling blocks for anticancer T cells. Trends Immunol (2012) 33(7):364–72. 10.1016/j.it.2012.02.006 22445288

[235] Redmond WL Sherman LA. Peripheral tolerance of CD8 T lymphocytes. Immunity (2005) 22(3):275–84. 10.1016/j.immuni.2005.01.010 15780985

[236] Cole DK Pumphrey NJ Boulter JM Sami M Bell JI Gostick E Human TCR-binding affinity is governed by MHC class restriction. J Immunol (2007) 178(9):5727–34. 10.4049/jimmunol.178.9.5727 17442956

[237] Schmid DA Irving MB Posevitz V Hebeisen M Posevitz-Fejfar A Sarria JC Evidence for a TCR affinity threshold delimiting maximal CD8 T cell function. J Immunol (2010) 184(9):4936–46. 10.4049/jimmunol.1000173 20351194

[238] Zehn D Bevan MJ. T cells with low avidity for a tissue-restricted antigen routinely evade central and peripheral tolerance and cause autoimmunity. Immunity (2006) 25(2):261–70. 10.1016/j.immuni.2006.06.009 16879996 PMC2774714

[239] Baumgaertner P Jandus C Rivals JP Derre L Lovgren T Baitsch L Vaccination-induced functional competence of circulating human tumor-specific CD8 T-cells. Int J Cancer (2012) 130(11):2607–17. 10.1002/ijc.26297 21796616

[240] Gubin MM Artyomov MN Mardis ER Schreiber RD. Tumor neoantigens: building a framework for personalized cancer immunotherapy. J Clin Invest (2015) 125(9):3413–21. 10.1172/JCI80008 26258412 PMC4588307

[241] Schumacher TN Schreiber RD. Neoantigens in cancer immunotherapy. Science (2015) 348(6230):69–74. 10.1126/science.aaa4971 25838375

[242] Twyman-Saint Victor C Rech AJ Maity A Rengan R Pauken KE Stelekati E Radiation and dual checkpoint blockade activate non-redundant immune mechanisms in cancer. Nature (2015) 520(7547):373–7. 10.1038/nature14292 25754329 PMC4401634

[243] Ochsenbein AF Klenerman P Karrer U Ludewig B Pericin M Hengartner H Immune surveillance against a solid tumor fails because of immunological ignorance. Proc Natl Acad Sci U S A (1999) 96(5):2233–8. 10.1073/pnas.96.5.2233 10051624 PMC26766

[244] Chu-Yuan H Jing P Yi-Sheng W He-Ping P Hui Y Chu-Xiong Z The impact of chemotherapy-associated neutrophil/lymphocyte counts on prognosis of adjuvant chemotherapy in colorectal cancer. BMC Cancer (2013) 13:177. 10.1186/1471-2407-13-177 23551939 PMC3621660

[245] Inoges S Rodriguez-Calvillo M Zabalegui N Lopez-Diaz de Cerio A Villanueva H Soria E Clinical benefit associated with idiotypic vaccination in patients with follicular lymphoma. J Natl Cancer Inst (2006) 98(18):1292–301. 10.1093/jnci/djj358 16985248

[246] Kakarla S Gottschalk S. CAR T cells for solid tumors: armed and ready to go? Cancer J (2014) 20(2):151–5. 10.1097/PPO.0000000000000032 24667962 PMC4050065

